# Non-aqueous electrowetting liquid lens with centimeter-level large aperture based on dielectric failure suppression principle

**DOI:** 10.1038/s41377-025-01777-2

**Published:** 2025-03-12

**Authors:** You-Ran Zhao, Zhao-Song Li, Yi Zheng, Di Wang, Xiao-Ke Lu, Yu-Cheng Lin, Hao-Ran Zhang, Chao Liu, Qiong-Hua Wang

**Affiliations:** School of Instrumentation and Optoelectronic Engineering, Beihang University, 100191 Beijing, China

**Keywords:** Applied optics, Optical techniques, Optical materials and structures

## Abstract

Liquid lens offers a novel approach to achieving large depth of field, wide viewing angle, high speed, and high-quality imaging in zoom optical systems. However, the aperture and reliability limit the lens’s performance in various optical applications. The liquid material is crucial for the reliability of the large-aperture liquid lens. To solve the dielectric failure problem associated with the large aperture, we first reveal the mechanism of dielectric failure based on the transport properties of electrolyte solutions and the impact of electrochemical reaction rates from physical chemistry so as to propose a theoretical method to suppress dielectric failure fundamentally. Based on this theory, we develop a series of non-aqueous organic solutions to suppress high-voltage dielectric failure. Next, we identify the optimal formulation for comprehensive optical performance and fabricate a centimeter-level large-aperture electrowetting liquid lens. This lens features an optical power variation range of −11.98 m^−1^ to 12.93 m^−1^, with clear and high-quality imaging function, which can enlarge the field of view and depth adjustment range of holographic reconstructions while maintaining excellent edge clarity of the reconstructed images. The proposed centimeter-level large-aperture non-aqueous electrowetting liquid lens effectively suppresses dielectric failure under high voltage, demonstrates excellent optical performance, and holds exciting potential for applications in 3D display, precision measurement, biomedical observation, and more.

## Abstract (web summary)

A non-aqueous electrowetting liquid lens with centimeter-level large aperture based on dielectric failure suppression principle is proposed.

## Graphical abstract

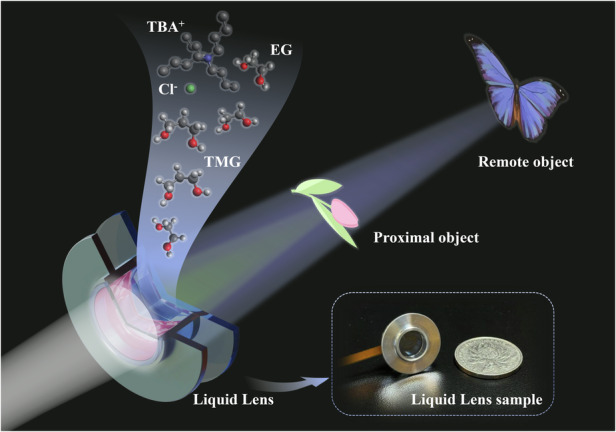

## Introduction

Liquid lens, as a novel type of adaptive zoom optical element, is widely used in fields such as biomedical detection^1–3^, in vivo cell observation^4–7^, 3D display^8–14^, and 3D reconstruction^15–17^, offering an advanced approach to achieving large depth of field, wide viewing angle, high speed, and high-quality imaging in zoom optical systems. As one of the most popular types of liquid lens, electrowetting liquid lens has advantages such as small size, easy integration, low power consumption, large zoom range, and fast response speed, being well-suited for compact and high-speed zoom optical imaging systems^12–15,18–21^. However, electrowetting liquid lens is prone to dielectric failure under high voltage, and with increasing aperture, the failure probability will be higher and occur within wider voltage range^22–29^. This reliability issue severely limits the application of large-aperture electrowetting liquid lens in various optical scenarios^30,31^.

Research on large-aperture electrowetting liquid lens is still ongoing, with most reported lens having apertures ranging from 3 mm to 6 mm^19^. Through extensive research, our team has increased the aperture of the electrowetting liquid lens to 8 mm to10 mm^14,22,32^. However, the reliability of the centimeter-level large-aperture liquid lens still needs to be improved further. The primary reason is that the area of the dielectric and hydrophobic layers increases with the aperture, extending the triple contact line, which requires higher chemical stability of the liquid^23^. Adjusting the types and concentrations of ions can enhance the reliability of electrowetting, but these studies focus on aqueous solutions and still face dielectric failure under high voltage^28,29,31,33^. The dielectric layer material exhibits time-dependent dielectric breakdown (TDDB) effects^34–36^. Multilayer dielectric structures, denser materials, and improved coating processes can effectively reduce the occurrence of dielectric failure^37–39^. However, using aqueous solutions still inevitably leads to dielectric failure, resulting in the generation of bubbles and damage that renders the lens unusable. Besides, larger apertures require larger areas of the dielectric coating, increasing the probability of depressions, voids, impurities, and uneven thickness^27,40^. The hydrophobic layer is typically composed of fluoropolymers, which preferentially adsorb anions. The production process of fluoropolymer hydrophobic layers involves solution vapor deposition, where solvent evaporation creates a porous structure. These pores and channels may provide pathways for cation or proton transfer, increasing the risk of aqueous solutions electrolysis^41,42^.

**Fig. 1:**
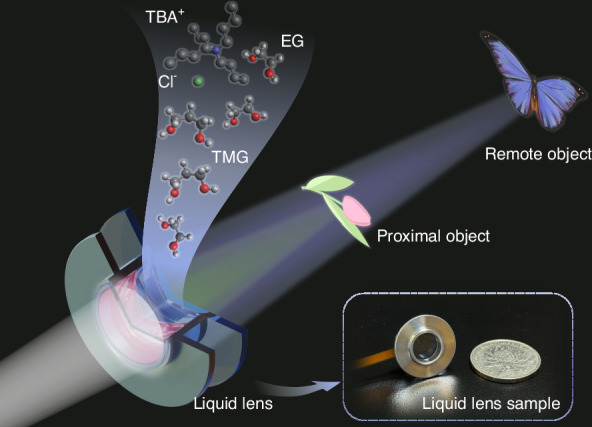
Concept diagram and sample of the proposed centimeter-level large-aperture non-aqueous electrowetting lens based on the principle of dielectric failure suppression

To address the issue of dielectric failure in large-aperture electrowetting liquid lens, we develop unique electrowetting liquid materials based on the theory of dielectric failure suppression. These materials are successfully applied to centimeter-level large-aperture electrowetting liquid lens, achieving ideal imaging results, as shown in Fig. 1. For the first time in this paper, the mechanisms of dielectric failure from the perspective of electrochemical reaction rates and the reactants and products of electrochemical reactions are analyzed, proposing a fundamental theoretical method to suppress dielectric failure. Guided by this mechanism, we develop a non-aqueous conductive liquid formulation to suppress dielectric failure and determine the optimal component proportion. Using standard coating processes and thicknesses for the dielectric and hydrophobic layers, we fabricate a centimeter-level electrowetting liquid lens that operates without dielectric failure under a high voltage of 200 V. The proposed liquid lens has the potential to become a core element in display technology, meeting the demand for a large field of view and long-term stable operation. To prove this point, the application of centimeter-level electrowetting liquid lens in holographic 3D display is demonstrated, significantly enhancing the field of view and depth adjustment range of the holographic images. The principle of dielectric failure suppression using non-aqueous electrowetting liquid proposed in this paper addresses the manufacturing bottleneck of large-aperture liquid lens. The developed liquid lens with high optical performance also holds promise for new applications in 3D display, precision measurement, and biomedical observation.

## Theory and principle

**Fig. 2:**
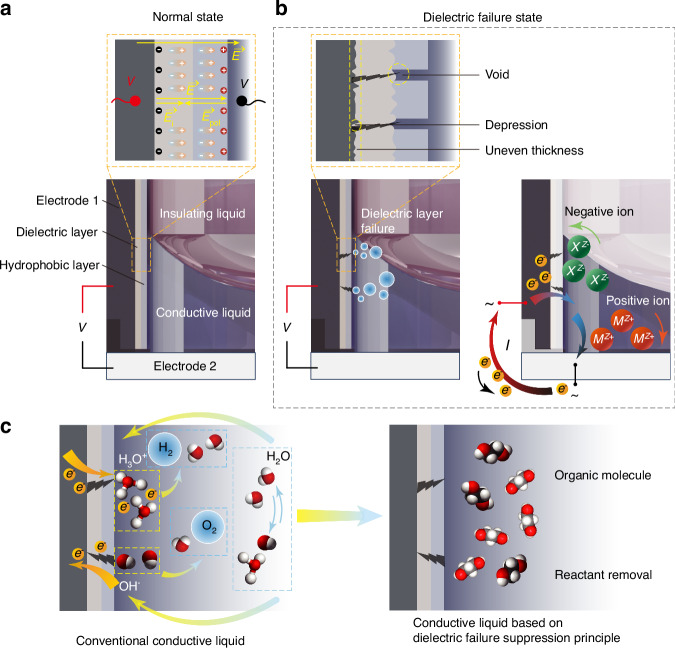
Principle of preparation of conductive liquid to suppress dielectric failure. **a** Schematic diagram showing the polarization of dielectric and hydrophobic layer materials under applied voltage in the normal state. **b** Schematic diagram of defects in the dielectric and hydrophobic layers and the dielectric failure current in the dielectric failure state. *M* is the positive ion group, *X* is the negative ion group, *Z* is the charge number, and *e*^–^ is electron. **c** Microscopic changes in the conventional conductive liquid and the conductive liquid based on the dielectric failure suppression principle during dielectric failure

The proposed theory of preparation of conductive liquid to suppress dielectric failure is illustrated in Fig. 2. Each component of the conductive liquid-hydrophobic layer-dielectric layer-electrode chain affects the response of the electrowetting element to electrical signals. Each component of this chain is interrelated, collectively impacting element reliability. This section first reveals the mechanism of dielectric failure by examining the properties of the dielectric layer and conductive liquid materials in the electric field throughout the entire circuit of an electrowetting liquid lens and the physical chemistry properties during the electrolysis reaction when the conductive liquid contacts the electrode after dielectric layer breakdown, as shown in Fig. 2a, b. Next, we reveal the principles for preparing dielectric failure suppression conductive liquid. These principles include the selection of raw materials based on the reactants and products of the electrochemical reaction and the proportions of raw materials based on the electrochemical reaction rate. Finally, a fundamental theoretical method is proposed to suppress dielectric failure, as shown in Fig. 2c.

### Mechanism revelation of dielectric failure based on electrochemistry approach

The electrowetting effect is described by the Young-Lippmann equation^19^, which gives the relationship between the applied voltage *V* and the contact angle *θ*_e_

1 $$\cos {\theta }_{{\rm{e}}}=\,\cos {\theta }_{0}+\frac{C}{2{\gamma }_{{\rm{ci}}}}{V}^{2}$$

where *θ*_0_ is the initial contact angle when no voltage is applied, *C* = *ε*_r_*ε*_0_ /*d* is the total capacitance per unit area of the dielectric and hydrophobic layers, *ε*_r_ is the total relative dielectric constant of the dielectric and hydrophobic layers, *ε*_0_ is the vacuum dielectric constant, *d* is the thickness of the dielectric layer, and *γ*_ci_ is the interfacial tension between the conductive phase and the insulating phase.

The circuit path of the electrowetting liquid lens consists of electrode-dielectric layer-hydrophobic layer-conductive liquid-electrode. When the conductive liquid undergoes normal electrowetting on the dielectric layer, opposite positive and negative charges accumulate on both sides of the dielectric layer. One side accumulates charges from contact with the electrode, and the other side accumulates charges from contact with the conductive liquid. Therefore, an electric field exists within the dielectric layer, as shown in Fig. 2a. The dielectric layer material is neither a perfect insulator nor a conductor. It cannot fully insulate between the conductive liquid and the electrode, and its molecules are not freely movable like electrons. However, under the influence of an external electric field $\overrightarrow{E}$, the molecules of the dielectric layer can become polarized, forming an internal electric field ${\overrightarrow{E}}_{{\rm{i}}}$ in conjunction with the polarization electric field ${\overrightarrow{E}}_{{\rm{pol}}}$ generated by the polarized charges at the two interfaces. According to electrodynamics principles, the numerical relationship between ${\overrightarrow{E}}_{{\rm{pol}}}$ and ${\overrightarrow{E}}_{{\rm{i}}}$ is as follows

2 $${E}_{{\rm{pol}}}=\frac{{\delta }_{{\rm{pol}}}}{{\varepsilon }_{0}}=-\chi {E}_{{\rm{i}}}$$

The direction of ${\overrightarrow{E}}_{{\rm{pol}}}$ is opposite to that of the internal electric field ${\overrightarrow{E}}_{{\rm{i}}}$, and *χ* is the dielectric susceptibility of the dielectric layer material. Therefore, the total internal electric field is partially shielded by the polarized charge, resulting in

3 $$\overrightarrow{E}=(1+\chi ){\overrightarrow{E}}_{{\rm{i}}}$$

Let ${\varepsilon }_{r}=1+\chi$ represent the relative dielectric constant. According to Eq. (3), polarized charges always counteract the external electric field, increasing in magnitude with the polarization rate. In the extreme case where the relative permittivity is sufficiently large, the internal electric field disappears, and the material behaves as a conductor. For an ideal dielectric layer, the electric field distribution is uniform. However, using the standard coating processes and coating with conventional thickness for electrowetting elements, the actual morphology of the dielectric layer is not uniformly smooth as assumed in the ideal case of a planar plate. It contains surface irregularities and internal voids where the membrane is thinner, resulting in denser electric field lines and higher potential differences. This leads to local field intensity and potential difference enhancement, which causes local fracture of the film layer, as shown in Fig. 2b.

The conductive liquid is another critical component in the circuit of the electrowetting liquid lens. The conductive liquid material typically comprises molecules with electric dipoles and has a relatively large relative permittivity. For example, at room temperature, the relative permittivities of water, propane-1,3-diol, and ethylene glycol are 78.5, 32, and 37, respectively. Polar molecules reorient under the electric field built by the external electrodes, thereby shielding a portion of the electric field created by the external electrodes. Meanwhile, ions in polar solutions move freely, accumulating at the electrodes of opposite charges until the external electric field is shielded, reaching equilibrium. There should be no current in the electrowetting circuit at equilibrium for direct current (DC) drive or alternating current (AC) drive without changing the current direction. Therefore, it is known that only non-equilibrium currents can pass through polar solutions, consisting of two parts: polarization current originating from polarized solvent molecules and current from solute ions migrating to the positive and negative electrodes. Both currents are almost transient. Direct current implies electrochemical reactions occurring on the electrodes, which must be in contact with the solution, as shown in Fig. 2b.

Concretely, in physical chemistry, the transport properties of electrolyte solutions explain the migration of ions under a potential gradient or electric field and their ability to conduct current. We reveal a method to suppress dielectric failure based on the transport properties of electrolyte solutions. In the electrowetting liquid lens, the conductive liquid is typically an electrolyte solution or a compound containing ions with free mobility. The conductivity *κ* of an electrolyte solution is defined as the solution’s conductance per unit area of the electrodes and per unit distance between the electrodes, as shown in Eq. (4)

4 $$\kappa ={Gl}/S$$

where *G* is the conductance, defined as the reciprocal of the resistance *R*, *l* is the length of the resistor, and *S* is the cross-sectional area of the resistor.

Furthermore, since *G* = *I*/*V*, where *I* is the electric current and *V* is the voltage in the circuit, the current density *j* = *I*/*S* and the electric field strength *E* = *V*/*l*, Eq. (4) can be rewritten as

5 $$\kappa =j/E$$

Therefore, conductivity can also be defined as the current density of a unit of electric field strength. When dielectric failure occurs, the electrolyte solution undergoes electrochemical reactions. Electrochemical reactions are accompanied by electric currents, and the reaction rate is directly proportional to the current. In electrochemistry, the rate of electrode reactions is expressed as

6 $${v}_{{\rm{cathode}}}=-\frac{j}{zF}$$

7 $${v}_{{\rm{anode}}}=\frac{j}{zF}$$

where *z* is the charge number, and *F* is the Faraday constant.

### Principle of conductive liquid preparation to suppress dielectric failure

Dielectric failure in electrowetting is particularly problematic because conventional aqueous solutions, once in contact with the electrode through a damaged dielectric layer, produce gas bubbles. An effective method to suppress dielectric failure should eliminate the adverse consequences following such failure. In this paper, we first apply the transport properties of electrolyte solutions in physical chemistry and the impact of electrochemical reaction rates to reveal the mechanism of dielectric failure. Consequently, a method to suppress dielectric failure is derived from this analysis, as shown in Fig. 2c.

From Eqs. (6)–(7), we derive an inference: a higher conductivity of the conductive liquid implies a greater propensity for intense electrode reactions. It must be noted that conductive liquids with high conductivity do not necessarily experience dielectric failure. However, once the liquid contacts the electrode, the electrolysis reactions of substances in the solution occur more quickly on the electrode in high-conductivity liquids compared to those with low conductivity. Additionally, it is crucial to remove reactive substances or ensure that the liquid cannot reach the electrode in the presence of voids, causing dielectric failure. From these perspectives, the consequences of dielectric failure are very subtle and unobservable because no electrochemical reaction will occur to produce gas bubbles, nor would any current be generated, thereby avoiding a significant voltage drop across the dielectric layer.

Consequently, we reveal the criteria for selecting the conductive liquid for the electrowetting lens: removing reactants involved in electrochemical reactions and controlling the conductivity of the liquid. We screen the electrolyte ions and solvent molecules contained in the electrowetting conductive liquid materials based on the conclusions derived above and the experimental foundation of our team’s previous research^22^. We select more stable, less prone to electrolysis, non-aqueous polar solvents, supplemented with a small number of organic salt ions to ensure that the solution has a certain conductivity, with a short characteristic relaxation time, resulting in an overall Ohmic response of the solution^28,43–45^. Additionally, considering the physical properties of the liquid material, such as density, refractive index, viscosity, etc., we utilize propane-1,3-diol(also known as trimethylene glycol, TMG) and ethylene glycol (EG) as solvents and tetrabutylammonium chloride (TBAC) as the solute. For specific details regarding material selection, please refer to Supplementary Material S1.

## Results

In this section, guided by the theory above, we develop a series of conductive liquids termed the EGG series. Firstly, we measure the basic physical properties of the EGG series liquids. Subsequently, we assess the electrowetting characteristics and dielectric failure characteristics of the EGG series liquids under applied voltage. Then, based on these properties and characteristics, we conduct a comprehensive evaluation to determine the optimal formulation and successfully fabricate a centimeter-level electrowetting liquid lens that suppresses dielectric failure, applied in holographic 3D displays.

### Physical properties of the EGG series liquids

**Fig. 3:**
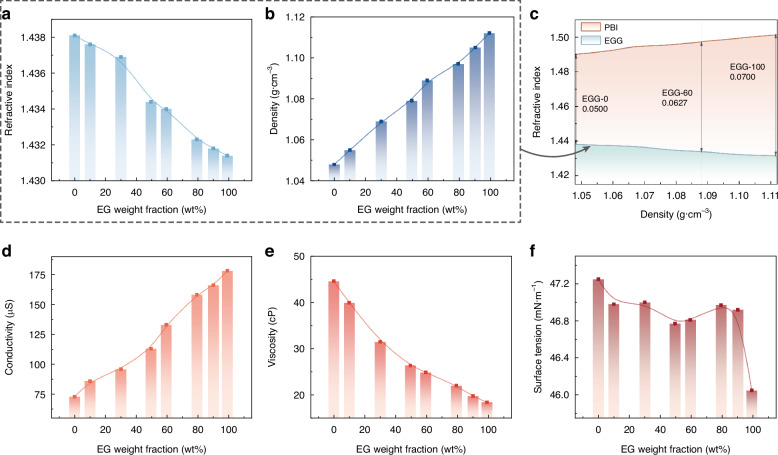
Parameters of the EGG series liquids. **a** Relationship between the EG weight fraction and the liquid refractive index. **b** Relationship between the EG weight fraction and the liquid density. **c** Relationship between the density and the refractive index of the biphasic liquids. **d** Relationship between the EG weight fraction and the liquid electrical conductivity. **e** Relationship between the EG weight fraction and the liquid viscosity. **f** Relationship between the EG weight fraction and the liquid surface tension

We prepare different ratios of EG and TMG as the conductive phase, denoted by EGG-wt, based on the weight fraction of EG, wt%. Each EGG-wt sample is supplemented with 1%wt of TBAC to enhance conductivity. The relationships between the tested liquid compositions and their refractive index, density, viscosity, surface tension, and conductivity are shown in Fig. 3. As the content of EG increases, there is a slight decrease in the refractive index of the conductive phase (only changing the third decimal place), a significant increase in density, an increase in conductivity, a decrease in viscosity, and almost no change in surface tension.

In electrowetting elements, a crucial relationship is the relationship between the density and refractive index of the liquid material due to the variation in the components of filled biphasic liquids. Therefore, the relationship between density and refractive index is illustrated visually in Fig. 3c. We also include the curve of the insulating phase density and refractive index in this graph. This helps to observe the refractive index difference Δ*n*_D_ better when densities are matched. We prepare the insulating phase PBI series liquids by mixing different proportions of ISOPAR^TM^ V fluid with p-bromoethylbenzene (PB) (Supplementary Material S2.1). It is observed that the contribution to the increased density when introducing EG can facilitate matching the conductive phase with a heavier insulating phase. This implies that the refractive index difference between the biphasic liquids can be significantly increased compared to when little or no EG is added (the refractive index difference between the EGG-100 and its density-matched insulating liquid is 1.4 times that of the EGG-0 and its matched insulating liquid). While the weight fraction of the solute in the conductive phase tested remains consistent, the conductivity increases with the proportion of EG, as mentioned above. Despite the absence of substances theoretically involved in electrochemical reactions in the conductive liquid composition, the hygroscopicity of EG and TBAC in the raw materials, as well as the inevitable impurities in the raw material synthesis, may introduce water into the conductive phase. Therefore, the increased EG concentration will increase the risk of dielectric failure to some extent.

### Electrowetting characteristics of the EGG series liquids

**Fig. 4:**
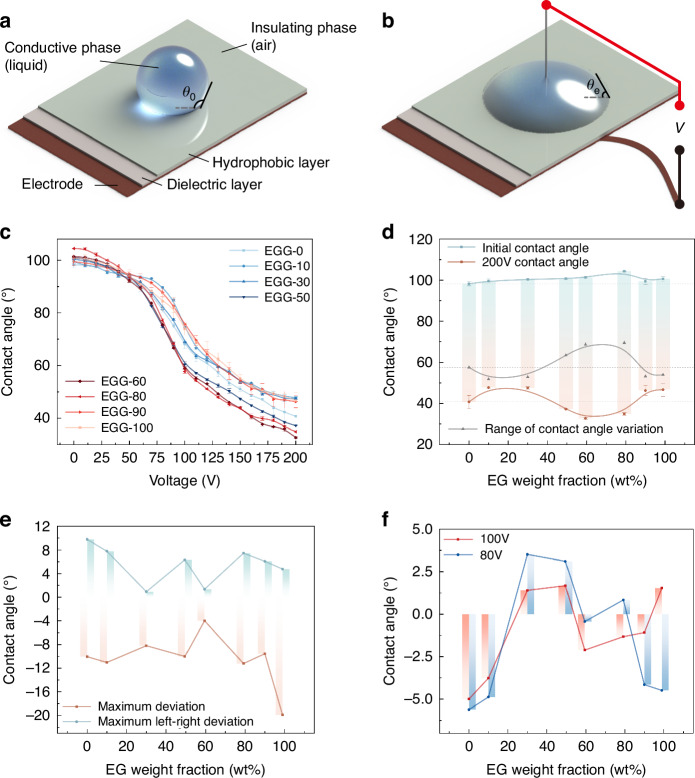
Schematic of the conventional electrowetting setup and the results of electrowetting contact angle measurements. **a** Electrowetting on the dielectric layer without voltage. **b** Electrowetting on the dielectric layer with voltage. **c** Relationship between the applied voltage and the contact angle of the droplet. **d** Relationship between the EG weight fraction and the initial contact angle, the contact angle at 200 V, and the range of contact angle of the droplet. **e** Relationship between the EG weight fraction and the maximum deviation and the maximum left-right deviation of the initial contact angle recovery. **f** Relationship between the EG weight fraction and the deviation of the contact angle at 80 V and 100 V

To balance the performance of the electrowetting element with the risk of dielectric failure, we conduct tests on the electrowetting properties of the proposed EGG series. The experimental setup is illustrated in Fig. 4a, b. We focus on differences before and after applying voltage to the conductive liquid, including the initial contact angle, the contact angle under different applied voltages, threshold voltage, and performances of dielectric failure. The relationship between contact angle and applied voltage is depicted in Fig. 4c. More detailed experimental data and theoretical values can be found in Supplementary Material S2.2.

The relationship between the applied voltage and the contact angle is processed and analyzed, as shown in Fig. 4d. The initial contact angle *θ*_0_ gradually increases as the EG content wt% ranges from 0% to 80% but decreases after reaching 90%. Across the entire range of applied voltages, the range of contact angle variation is inferior for EG content with wt%<50% and wt%>80% compared to that without EG, despite the larger initial contact angles observed in this range. Specifically, the contact angle variations of *θ*_EGG-10_ (51.9°), *θ*_EGG-30_ (52.8°), *θ*_EGG-90_ (53.2°), and *θ*_EGG-100_ (54°) are all less than *θ*_EGG-0_ (57.6°). Conversely, for EG content wt% ranging from 50% to 80%, there is a significant increase in the range of contact angle variation compared to that without EG, with the contact angle variations of *θ*_EGG-50_ (63.6°), *θ*_EGG-60_ (68.7°), and *θ*_EGG-80_ (69.6°) exceeding that of *θ*_EGG-0_.

Furthermore, regarding the threshold voltage and dielectric failure, across a 3μm dielectric layer, the threshold voltage for the EGG series is consistently below 20 V. With EG content ranging between 0% ≤ wt% ≤ 80%, no dielectric failure occurs under an effective voltage of 200 V. However, for the EGG-90, dielectric failure occurs with an applied voltage of 120 V. For the EGG-100, dielectric failure occurs with an applied voltage of 140 V.

### Dielectric failure characteristics of the EGG series liquids

We conduct several experiments to verify the dielectric failure suppression capability of the EGG series liquids and characterize the dielectric failure observed in the EGG-90 and EGG-100. First, based on the proposed theory and the material composition of the EGG series liquids, we provide the possible electrochemical reaction equations after dielectric failure by measuring the pH values. Next, we perform droplet cyclic voltage application tests, record the changes in the electrowetting contact angles of the EGG series droplets, and analyze the experimental phenomena and the impact of dielectric failure on the EGG series liquids. The comprehensive performance of the EGG series in electrowetting characteristic tests confirms the dielectric failure suppression effect of the EGG series liquids.

The components of the conductive phase are all organic substances. After forming the solution, the electrolyte TBAC, as a strong electrolyte, will completely dissociate, forming positively charged tetra-n-butylammonium cations (TBA^+^) and negatively charged chloride ions (Cl^-^). Among them, both EG and TBAC are prone to introduce water as impurities into the prepared solution. Previous studies on non-aqueous solvents have demonstrated that organic molecules and TBA^+^ ions do not participate in electrolysis or induce more severe electrolysis^28^. Therefore, when analyzing the electrochemical reactions after dielectric failure, we consider the inert electrode reaction of water molecules and chloride ions in the electrolytic cell (the analysis is detailed in Supplementary Material S2.3).

To verify our hypothesis regarding the electrochemical reactions in the liquid lens after dielectric failure, we conduct pH tests on the conductive-phase liquid post-dielectric failure and compare them with the original pH values of pure water, the conductive liquid, and the insulating liquid (Supplementary Material S2.3, Fig. S4). Before dielectric failure occurs, the pH values of the conductive and insulating liquids approximate that of pure water. After dielectric failure, the pH value of the conductive liquid ranges between 7.7 and 7.9, indicating a slight increase, suggesting weak alkalinity in the solution. This rise in pH value implies an increase in OH^-^ concentration in the conductive liquid after dielectric failure. Therefore, we hypothesize that the electrochemical reaction occurring during dielectric failure should be:

$${\rm{At\; cathode}}:2{{\rm{H}}}_{2}{\rm{O}}+2{{\rm{e}}}^{-}\to {{\rm{H}}}_{2}\uparrow +{2{\rm{OH}}}^{-}$$

$${\rm{At\; anode}}:{2{\rm{Cl}}}^{-}\to {{\rm{Cl}}}_{2}\uparrow +{2{\rm{e}}}^{-}$$

$${\rm{Net\; reaction}}:{2{\rm{H}}}_{2}{\rm{O}}+{2{\rm{Cl}}}^{-}\to {{\rm{H}}}_{2}\uparrow +{{\rm{Cl}}}_{2}\uparrow +{2{\rm{OH}}}^{-}$$

The increase in pH value leads to a decrease in the surface tension of the solution^46^. The surface tension of the EGG-100 after dielectric failure is measured to be 44.65 mN m^−1^, significantly lower than the surface tension before dielectric failure of 46.05 mN m^−1^. Unlike the liquids of conventional liquid lens, in the EGG series liquids, water exists only as trace impurities. Therefore, electrochemical reactions do not continue indefinitely. Once the trace water has reacted completely, the electrochemical reaction ceases.

The experiments above demonstrate that the EGG-80 serves as the critical component ratio. The EGG series liquids adjust the conductivity by varying the EG concentration, thereby avoiding bubble formation due to dielectric failure under high voltage. To further validate the suppression effect of dielectric failure by the EGG series liquids, we conduct multiple cyclic voltage application tests on the contact angle measurements of the EGG series liquids. We measure the recovery of the initial contact angle of the droplets and the consistency of the contact angle at 80 V and 100 V. The recovery of the initial contact angle is characterized by the maximum deviation of the initial contact angle after multiple voltage removals and the maximum left-right deviation in a single measurement. The consistency of the contact angle at 80 V and 100 V is characterized by the maximum deviation of the contact angle after applying the voltage multiple times at these voltages (Supplementary Material S2.3). The experimental results are shown in Fig. 4e, f. The experiments demonstrate that for the recovery deviation of the initial contact angle, the EGG-60 performs the best with a maximum deviation of about −4° and a maximum left-right deviation of 1.3°. The EGG-100 has the largest recovery deviation of the initial contact angle, decreasing by 19.9°. For the consistency of contact angles at 80 V and 100 V, all liquids in the EGG series liquids exhibit good consistency, with deviations ranging from −5.6° to +3.5°, including the EGG-90 and EGG-100, which experience dielectric failure. The experimental results demonstrate the feasibility of our inference: liquids with higher conductivity have faster electrochemical reaction rates after dielectric failure, leading to more severe phenomena. By controlling the concentration of EG in the EGG series liquids, we manage the conductivity so as to determine the specific component ratios of the EGG series liquids that possess dielectric failure suppression properties.

Finally, we analyze the impact of dielectric failure on the EGG series liquids used in the experiments. For the EGG series liquids developed in this paper, only EGG-90 and EGG-100 prepared with extremely high concentrations of EG experience dielectric failure. Even when the dielectric failure occurs, the raw materials involved in the electrochemical reaction are not the main components of the EGG series liquids. Consequently, dielectric failure does not persist, and its effects are milder and more controllable compared to conventional aqueous conductive liquids. When no voltage is applied, the initial contact angle decreases due to reduced surface tension. When voltage is applied, the ability to change the contact angle remains almost unchanged due to the minimal variation in the composition of the conductive liquid.

Based on all the tests above, we select the most ideal composition ratios in the EGG series liquids, namely EGG-50 to EGG-80. Within this concentration range, the refractive index of the conductive liquid is relatively small, the density can help the conductive liquid to match insulating liquid with a larger refractive index difference from it, and the viscosity is between 20 cP and 30 cP, which is more suitable. Furthermore, the electrowetting characteristics of the EGG-50, EGG-60, and EGG-80 are excellent, with the large initial contact angle, the wide range of contact angle variation, and no occurrence of dielectric failure.

### Fabrication of the EGG series electrowetting liquid lenses

**Fig. 5:**
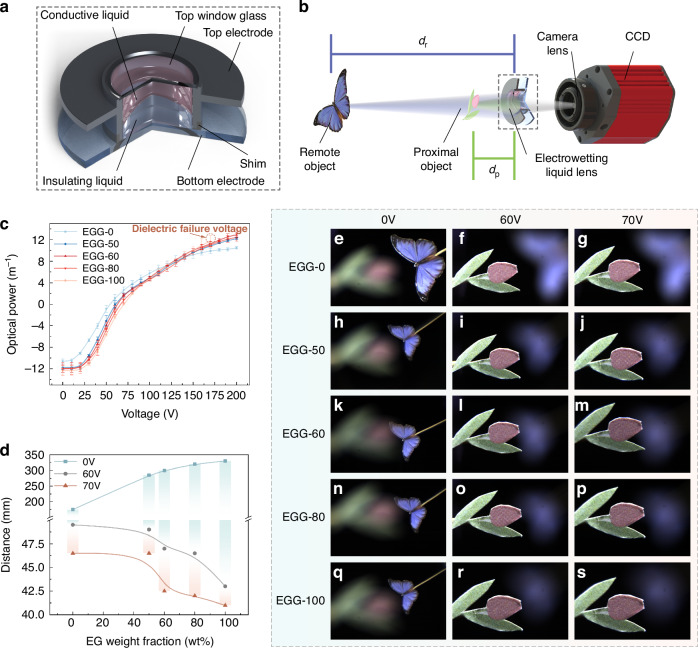
Optical performance test of the EGG series electrowetting liquid lenses. **a** Structure of the EGG series electrowetting liquid lens. **b** Setup of the imaging experiment. **c** Optical power of the EGG series liquid lenses. **d** Relationship between the EG weight fraction and the object distance with applied voltages of 0 V, 60 V, and 70 V. **e**–**s** Imaging experiment results of the EGG series electrowetting liquid lenses

The schematic structure of the proposed 10 mm-aperture EGG series electrowetting liquid lens is illustrated in Fig. 5a. The lens comprises an upper electrode, a lower electrode, a shim, an upper window glass, the conductive liquid, and the insulating liquid. Through simulations, we optimize the thickness of the liquid lens to achieve a large aperture with a relatively small thickness, which enhances the field of view when it is applied to imaging systems. The lens has an aperture of 10 mm and a thickness of only 6 mm, resulting in an aperture-thickness ratio of 1.67. Details of the simulation, design, and fabrication of the lens are detailed in Supplementary Material S3.1.

### Performance experiment of the EGG series electrowetting liquid lenses

Based on the above analysis, we select the best-performing EGG-50 and EGG-60 conductive liquids, as well as the critical EGG-80, to fill in the lenses with PBI series liquids with matching density as the insulating liquid. Additionally, we produce two additional lenses using the EGG-0 and EGG-100 as controls. Detailed parameters for fabricating the five lenses and the liquids are provided in Supplementary Material S3.2, Table S1. The optical performance of the five lenses, including optical power, zoom performance, and imaging performance, are measured. Figure 5b illustrates the optical path of the imaging experiment. The results of the optical power measurements, the zoom performance, and the imaging performance are shown in Fig. 5c, Fig. 5d, and Fig. 5e–s, respectively.

The relationship between the optical power of the lens and the applied voltage, ranging from 0 V to 200 V, tested using an auto lensmeter (Type of COT-L800, Shanghai Chang’E Optical Equipment & Instrument Technology Co., Ltd., China), is shown in Fig. 5c. The initial optical powers of the lenses filled with the EGG-0, EGG-50, EGG-60, EGG-80, and EGG-100 are −10.59 m^−1^, −11.78 m^−1^, −11.98 m^−1^, −12.02 m^−1^, and −12.35 m^−1^, respectively. When the optical power of the five liquid lenses is 0 m^−1^, the applied voltages increase slightly with increasing EG concentration, reaching 54 V, 60 V, 63 V, 67 V, and 70 V, respectively. At 170 V, the optical powers of the five liquid lenses are 9.92 m^−1^, 10.84 m^−1^, 11.24 m^−1^, 11.03 m^−1^, and 11.70 m^−1^, respectively, with the lens filled with the EGG-100 experiencing dielectric failure. At 200 V, the lenses filled with the EGG-0, EGG-50, EGG-60, and EGG-80 still do not experience dielectric failure. The optical powers of the four liquid lenses are 10.49 m^−1^, 12.21 m^−1^, 12.93 m^−1^, and 12.45 m^−1^, respectively. Within the applied voltage range, the maximum optical powers variation range achieved by the five liquid lenses are 21.08 m^−1^, 23.99 m^−1^, 24.91 m^−1^, 24.47 m^−1^, and 24.05 m^−1^, respectively. It is worth noting that after a certain period following dielectric failure in the liquid lens filled with the EGG-100, no bubbles are observed on the two electrodes of the liquid lens, and the liquid lens still maintains its focusing ability.

In the imaging experiment, the experimental optical path consists of a CCD and camera lens, the EGG series liquid lens, a proximal object, and a remote object, forming a zoom camera system, as shown in Fig. 5b. The positions of the liquid lens and the CCD camera remain fixed while the object is finely adjusted using a servo motor translation stage to adjust the object distance. By changing the voltage applied to the liquid lens, the lens focuses on the objects at different distances. Due to the limited displacement range of the translation stage, the applied voltage range is from 0 V to 70 V. Figure 5d shows the change in object distance for each of the five liquid lenses at applied voltages of 0 V, 60 V, and 70 V. Figure 5e-s present the imaging experiment results of the zoom camera system using the five liquid lenses respectively. As the EG content increases, the zoom range of the lenses expands. In the zoom camera system, when applying voltages from 0 V to 70 V, the zoom ranges of the five liquid lenses are 128.5 mm, 238.5 mm, 257.5 mm, 278 mm, and 289 mm, respectively. The imaging results in Fig. 5e-s indicate that all five liquid lenses exhibit excellent image quality with clear and low aberration. Details of the imaging experiments are provided in Supplementary Material S3.2, Figs. S10 and S11.

Moreover, the response time of the five lenses is measured (Supplementary Material S3.2, Fig. S9). The EGG-60 lens exhibits a relatively fast response time, with a rise response time of 174 ms and a fall response time of 45 ms. Through liquid lens performance testing, the EGG-60 exhibits the best overall performance. The liquid lens with the EGG-60 has the largest optical power range and maintains a relatively fast response time under a 10 mm aperture. Therefore, we conduct an application experiment using the electrowetting liquid lens using the EGG-60.

Compared with elastic film liquid lens, electrowetting liquid lens has significant advantages. Elastic film lens usually contains only a single liquid, and the effect of gravity becomes more obvious with the increase of aperture size. In contrast, the electrowetting liquid lens utilizes density-matched biphasic liquids, which effectively reduces the gravity effect. Besides, mechanically-driven elastic film lens often requires large volume and the focusing speed is slow. Piezoelectrically-driven elastic film lens generally provides a limited focal range. Electromagnetically-driven elastic film liquid lens that requires current to operate typically exhibits high power consumption and significant heat generation, which impairs focusing stability. Therefore, electrowetting liquid lens has certain advantages in large focal range, faster response speed, low power consumption and easy integration.

### Application experiment of the EGG-60 electrowetting liquid lens

**Fig. 6:**
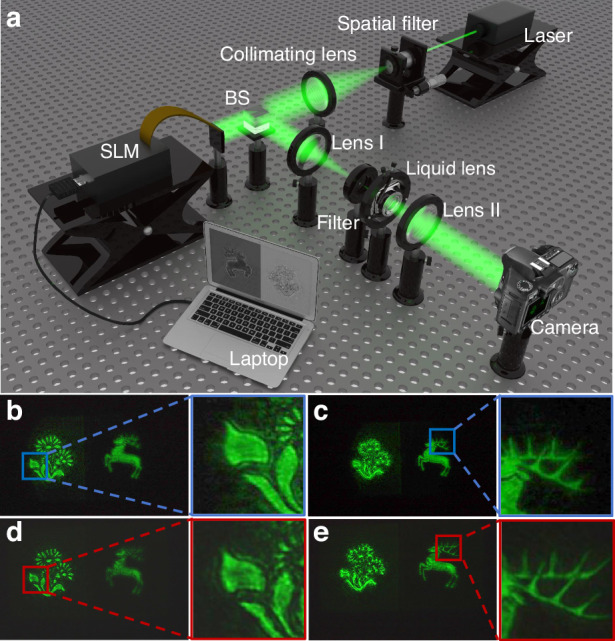
Application experimental system and experimental results. **a** Schematic structure of the liquid lens-based holographic 3D display system. **b**, **c** Experimental effects of focusing the “flower” and “deer” using the commercial A-39N0 liquid lens, respectively. **d**, **e** Experimental effects of focusing the “flower” and “deer” using the proposed EGG-60 liquid lens, respectively

To verify the advantages of the proposed liquid lens in the application, a 3D display experiment is carried out. A holographic 3D display system based on the liquid lens is built to adjust the reconstruction position of the holographic reconstructed images dynamically. The schematic structure of the system is shown in Fig. 6a. The system is composed of a laser, a spatial filter, a collimating lens, a beam splitter (BS), a spatial light modulator (SLM), a 4*f* system (including filter, lens I and lens II), a designed liquid lens with the EGG-60 and a laptop. The wavelength of the laser is 532 nm. The spatial filter and collimating lens are used to generate uniform parallel light. The resolution of the SLM produced by Xi’an CAS Microstar Technology Co is 1920×1080, and the pixel pitch is 6.4 μm. The 4f system is used to filter out the stray light. Both lens I and lens II have a focal length of 200 mm. The liquid lens is located 50 mm behind the filter. The camera used to capture the holographic reconstructed images is a Canon EOS 6D Mark II. The distance between the camera and Lens II is 240 mm. The laptop is used to load the hologram and drive the liquid lens. By changing the driving voltage of the liquid lens, holographic reconstructed images with different reconstruction distances can be captured by the camera.

In the comparative experiment, the commercial A-39N0 liquid lens produced by Corning®-Varioptic® (the aperture is 3.9 mm) and the proposed EGG-60 liquid lens (the aperture is 10 mm) are used. Objects “flower” and “deer” in two different depths are used as a recorded 3D object. The hologram of the object is calculated by using the physical model-driven network^47^. The recording distances of “flower” and “deer” are 160 mm and 200 mm, respectively. The resolution of the hologram is 1920 × 1072. The effect of the holographic reconstructed images when using different liquid lenses is shown in Fig. 6b-e. Comparing Fig. 6b and Fig. 6d, it can be found that compared with the commercial A-39N0 liquid lens, the proposed EGG-60 lens can significantly reduce the generation of stray light and improve the quality of the holographic reconstructed image. Meanwhile, since the proposed EGG-60 lens has more advantages in aperture, more details of the holographic reconstructed image can be observed, such as the complete antlers, as shown in Fig. 6c and Fig. 6e. In order to compare the results more intuitively, we enlarge the details in the box in Fig. 6. Obviously, with the proposed EGG-60 lens, the edge of the “flower” and the horn of the “deer” can be well reconstructed. However, the reconstructed image using the commercial A-39N0 liquid lens is incomplete. The experiments show that the EGG-60 liquid lens is more advantageous in adjusting the reconstruction position and maintaining the quality of the holographic reconstructed image. More analysis of the experimental results can be found in Supplementary Material S3.3.

## Discussion

This study pioneers the application of electrolyte solution transport properties and electrochemical reaction rates from physical chemistry to reveal the mechanisms of dielectric failure. By understanding the electrochemical origins of dielectric failure, we identify a theoretical approach to completely suppressing it. Our innovative method involves removing reactants and controlling the conductivity of the conductive liquid, leading to the successful development of a nonaqueous conductive liquid that can withstand high voltages up to 200 V without dielectric failure. Using the optimized EGG-60 composition, we fabricate a 10 mm electrowetting liquid lens. This lens exhibits excellent optical performance, with a maximum focal power change of 24.91 m^−1^, a rise time of 174 ms, and a fall time of 45 ms, producing clear and high-quality imaging function. Experiments demonstrate that the 10 mm EGG-60 electrowetting liquid lens can flexibly adjust the reconstruction position of the holographic images in holographic 3D display technology while maintaining exceptional edge sharpness. The EGG series nonaqueous electrowetting liquids introduced in this paper achieve outstanding dielectric failure suppression, breaking through the fabrication barriers of a centimeter-level large-aperture lens. This advancement holds the potential to revolutionize fields such as 3D display and reconstruction, biomedical observation, and more, offering unprecedented capabilities and new possibilities.

## Materials and methods

### Details of droplet experiment

As shown in Fig. 4a, b, a dielectric and a hydrophobic layer are sequentially deposited on a planar electrode. A droplet of conductive phase liquid is placed on the hydrophobic layer. When a voltage *V* is applied between the droplet and the electrode, the droplet is spread out, causing the contact angle *θ*_0_ between the droplet and the hydrophobic layer to decrease to *θ*_e_, as determined by Eq. (1). In our experimental setup, the planar electrode is made of copper, the dielectric layer is coated with 3 μm of ParyleneC, and the hydrophobic layer is coated with ~100 nm of Teflon AF2400. The insulating phase is air. The conductive phase is the EGG series liquids.

### Training details

The DIV2K dataset is used for training the PMD-Net. The PMD-Net is built based on the PyTorch deep learning framework. AdamW optimization algorithm is used to train the PMD-Net. PyCharm integrated development tool is used as the platform to build the PMD-Net. The number of training epochs for the PMD-Net is 200, and the batch size is 1. The PMD-Net is deployed on a laptop equipped with an Intel Core i7-11800H CPU and an NVIDIA GeForce RTX 3080 GPU.

## Supplementary information

SUPPLEMENTAL MATERIAL

## Data Availability

The corresponding author can provide the supporting data upon reasonable request.

## References

[1] Shi, R. H. et al. Random-access wide-field mesoscopy for centimetre-scale imaging of biodynamics with subcellular resolution. *Nat. Photonics* **18**, 721–730 (2024).

[2] Zhang, R. N. et al. High‐speed multi‐modal extended depth‐of‐field microscopy with an electrically tunable lens. *Laser Photonics Rev.* **18**, 2300770 (2024).

[3] Xie, H. et al. Multifocal fluorescence video-rate imaging of centimetre-wide arbitrarily shaped brain surfaces at micrometric resolution. *Nat. Biomed. Eng.* **8**, 740–753 (2024). 38057428 10.1038/s41551-023-01155-6 PMC11250366

[4] Qian, J. M. et al. Structured illumination microscopy based on principal component analysis. *eLight* **3**, 4 (2023). 10.1364/OL.480330 36563399

[5] Zong, W. J. et al. Miniature two-photon microscopy for enlarged field-of-view, multi-plane and long-term brain imaging. *Nat. Methods* **18**, 46–49 (2021). 33408404 10.1038/s41592-020-01024-z

[6] Li, D. Y. et al. A Through-Intact-Skull (TIS) chronic window technique for cortical structure and function observation in mice. *eLight* **2**, 15 (2022).

[7] Xu, C. et al. Liquid-shaped microlens for scalable production of ultrahigh-resolution optical coherence tomography microendoscope. *Commun. Eng.* **3** (2024).

[8] Chen, J. et al. Design of foveated contact lens display for augmented reality. *Opt. Express* **27**, 38204–38219 (2019). 31878591 10.1364/OE.381200

[9] Xiong, J. H. & Wu, S. T. Planar liquid crystal polarization optics for augmented reality and virtual reality: from fundamentals to applications. *eLight* **1**, 3 (2021).

[10] Wang, D. et al. Holographic capture and projection system of real object based on tunable zoom lens. *PhotoniX* **1**, 6 (2020).

[11] Ding, Y. Q. et al. Waveguide-based augmented reality displays: perspectives and challenges. *eLight* **3**, 24 (2023).

[12] Hayes, R. A. & Feenstra, B. J. Video-speed electronic paper based on electrowetting. *Nature* **425**, 383–385 (2003). 14508484 10.1038/nature01988

[13] Heikenfeld, J. et al. Electrofluidic displays using Young–Laplace transposition of brilliant pigment dispersions. *Nat. Photonics* **3**, 292–296 (2009).

[14] Wang, D. et al. Liquid lens based holographic camera for real 3D scene hologram acquisition using end-to-end physical model-driven network. *Light Sci. Appl.* **13**, 62 (2024). 38424072 10.1038/s41377-024-01410-8 PMC10904790

[15] Liu, C. et al. Continuous optical zoom microscope with extended depth of field and 3D reconstruction. *PhotoniX* **3**, 20 (2022).

[16] Petsch, S. et al. The engineered eyeball, a tunable imaging system using soft-matter micro-optics. *Light Sci. Appl.* **5**, e16068 (2016). 30167172 10.1038/lsa.2016.68 PMC6059944

[17] He, C., Antonello, J. & Booth, M. J. Vectorial adaptive optics. *eLight* **3**, 23 (2023).

[18] Zhou, G. M. et al. Electrotunable liquid sulfur microdroplets. *Nat. Commun.* **11**, 606 (2020). 32001696 10.1038/s41467-020-14438-2 PMC6992759

[19] Liu, C. et al. Tunable liquid lenses: emerging technologies and future perspectives. *Laser Photonics Rev.* **17**, 2300274 (2023).

[20] Chen, S. H. et al. Optofluidics in bio-imaging applications. *Photonics Res.* **7**, 532–542 (2019).

[21] Cheng, Y., Cao, J. & Hao, Q. Optical beam steering using liquid-based devices. *Opt. Lasers Eng.* **146**, 106700 (2021).

[22] Xu, J. B. et al. Non-aqueous organic solution based on a large-aperture spherical electrowetting liquid lens with a wide tunable focal length range. *J. Mater. Chem. C* **10**, 6778–6793 (2022).

[23] Zhao, Y. R. et al. Tunable optofluidic Fresnel lens with ring-shaped electrodes. *Opt. Lasers Eng.* **176**, 108087 (2024).

[24] Mibus, M. et al. Failure modes during low-voltage electrowetting. *ACS Appl. Mater. Interfaces* **8**, 15767–15777 (2016). 27253515 10.1021/acsami.6b02791

[25] Cao, J. P. et al. Replaceable dielectric film for low-voltage and high-performance electrowetting-based digital microfluidics. *Langmuir* **39**, 10189–10198 (2023). 37432677 10.1021/acs.langmuir.3c01098

[26] Papaderakis, A. A. et al. Deciphering the mechanism of electrowetting on conductors with immiscible electrolytes. *Electrochim. Acta* **452**, 142342 (2023).

[27] Zhou, R. et al. Experimental study on the reliability of water/fluoropolymer/ITO contact in electrowetting displays. *Results Phys.* **12**, 1991–1998 (2019).

[28] Raj, B. et al. Ion and liquid dependent dielectric failure in electrowetting systems. *Langmuir* **25**, 12387–12392 (2009). 19678654 10.1021/la9016933

[29] Dhindsa, M., Kuiper, S. & Heikenfeld, J. Reliable and low-voltage electrowetting on thin parylene films. *Thin Solid Films* **519**, 3346–3351 (2011).

[30] Fan, M. Y. et al. Effect of liquid conductivity on optical and electric performances of the electrowetting display system with a thick dielectric layer. *Results Phys.* **16**, 102904 (2020).

[31] Li, J. et al. Ionic-surfactant-mediated electro-dewetting for digital microfluidics. *Nature* **572**, 507–510 (2019). 31435058 10.1038/s41586-019-1491-x

[32] Xu, J. B. et al. Triple-layer spherical electrowetting liquid lens with large-aperture and high zoom ratio. *Opt. Lasers Eng.* **160**, 107311 (2023).

[33] Papaderakis, A. A. et al. Taming electrowetting using highly concentrated aqueous solutions. *J. Phys. Chem. C* **126**, 21071–21083 (2022). 10.1021/acs.jpcc.2c06517 PMC9761672 36561202

[34] Van Der Holst, J. J. M. et al. Monte Carlo study of charge transport in organic sandwich-type single-carrier devices: effects of coulomb interactions. *Phys. Rev. B* **83**, 085206 (2011).

[35] Padovani, A. et al. A microscopic mechanism of dielectric breakdown in SiO_2_ films: an insight from multi-scale modeling. *J. Appl. Phys.* **121**, 155101 (2017).

[36] Zhang, Q. Q. et al. Three-dimensional mechanistic modeling of time-dependent dielectric breakdown in polycrystalline thin films. *Phys. Rev. Appl.* **19**, 024008 (2023).

[37] Dhindsa, M. et al. Electrowetting without electrolysis on self-healing dielectrics. *Langmuir* **27**, 5665–5670 (2011). 21456569 10.1021/la1051468

[38] Schultz, A. et al. Detailed analysis of defect reduction in electrowetting dielectrics through a two-layer ‘barrier’ approach. *Thin Solid Films* **534**, 348–355 (2013).

[39] Grisatya, A. & Won, Y. H. Multi-layer insulator for low voltage and breakdown voltage enhancement in electrowetting-on-dielectric. *Proc. SPIE* **8987**, 89871S.

[40] Ramachandran, A. et al. A study of parylene c polymer deposition inside microscale gaps. *IEEE Trans. Adv. Packaging* **30**, 712–724 (2007).

[41] Zimmermann, R., Rein, N. & Werner, C. Water ion adsorption dominates charging at nonpolar polymer surfaces in multivalent electrolytes. *Phys. Chem. Chem. Phys.* **11**, 4360–4364 (2009). 19458839 10.1039/b900755e

[42] Koo, B. & Kim, C. J. Evaluation of repeated electrowetting on three different fluoropolymer top coatings. *J. Micromech. Microeng.* **23**, 067002 (2013).

[43] Chatterjee, D. et al. Droplet-based microfluidics with nonaqueous solvents and solutions. *Lab Chip* **6**, 199–206 (2006). 16450028 10.1039/b515566e

[44] Chevalliot, S. et al. Analysis of nonaqueous electrowetting fluids for displays. *J. Disp. Technol.* **7**, 649–656 (2011).

[45] Zhang, Y. et al. Density, viscosity, surface tension of 1,3-propanediol + dimethyl sulfoxide mixed solutions and their intermolecular forces from spectra and computational chemistry. *Fluid Phase Equilibria* **534**, 112965 (2021).

[46] Akhlaghi, N., Riahi, S. & Parvaneh, R. Interfacial tension behavior of a nonionic surfactant in oil/water system; salinity, pH, temperature, and ionic strength effects. *J. Pet. Sci. Eng.* **198**, 108177 (2021).

[47] Wang, D. et al. High-quality holographic 3D display system based on virtual splicing of spatial light modulator. *ACS Photonics* **10**, 2297–2307 (2023).

