# Supplementary material for: Non-aqueous electrowetting liquid lens with centimeter-level large aperture based on dielectric failure suppression principle
Source: Light Sci Appl. 2025 Mar 12;14:120. doi: 10.1038/s41377-025-01777-2 (PMC11897351; doi:10.1038/s41377-025-01777-2)
Supplement: Supplementary file 1 — SUPPLEMENTAL MATERIAL [file 41377_2025_1777_MOESM1_ESM.docx]

SUPPLEMENTARY MATERIAL

Non-aqueous electrowetting liquid lens with centimeter-level large aperture based on dielectric failure suppression principle

You-Ran Zhao, Zhao-Song Li, Yi Zheng, Di Wang, Xiao-Ke Lu, Yu-Cheng Lin,

Hao-Ran Zhang, Chao Liu^*^, and Qiong-Hua Wang^*^

*School of Instrumentation and Optoelectronic Engineering, Beihang University, Beijing 100191, China.*

**Correspondence: C Liu, E-mail: chaoliu@buaa.edu.cn;*

*QH Wang, E-mail:* [*qionghua@buaa.edu.cn*](mailto:qionghua@buaa.edu.cn)

16 pages, 12 figures S1-S12

**S1 Additional details on principle**

Unlike metal conductors, electrolyte solutions conduct electricity through ions carrying positive and negative charges. The contribution to conductivity varies among different ions and solvent environments, which is expressed as the ion mobility *u_B_*. The ion mobility *u_B_* is defined as the ion's migration velocity under a unit electric field strength

| $u_{B}=\frac{\upsilon_{B}}{E}$ | (S1) |
| --- | --- |

where *B* represents the cation or anion, and *υ_B_* is the ion migration velocity.

Let the electrolyte be $M_{\nu_{+}}X_{\nu_{-}}$, with a molar concentration *c* and dissociation degree *α*, dissociating according to the following equation

| $M_{\nu_{+}}X_{\nu_{-}}\rightleftharpoons\nu_{+}M^{z_{+}}+\nu_{-}X^{z_{-}}$ | (S2) |
| --- | --- |

where *ν_B_* is the stoichiometric number of ions. Due to the electro-neutrality of the electrolyte solution, $\nu_{+}z_{+}=\nu_{-}\left|z_{-}\right|$.

Considering any cross-section of area *S* in the conductivity cell over a time interval *τ*, the volume on one side of this section with a distance of *υ*_+_*τ* from the section is *υ*_+_*τS*. The positive ions within this volume can pass through the section in *τ*. The number of these ions is *υ*_+_*τScαν*_+_, and the charge carried by these ions is *υ*_+_*τScαν*_+_·*z*_+_*F*. Similarly, on the other side of this section, with a distance of *υ*-*τ*, the volume is *υ*-*τS*. The negative ions within this volume can pass through the section in *τ*. The number of these ions is *υ*-*τScαν*-, and the charge carried by these ions is *υ*-*τScαν*-·|*z*-|*F*. The charge passing through the section per unit of time is the current, so the current constituted by the positive and negative charges is as follows

| $\begin{matrix} I=I_{+}+I_{-} \\ =Sc\alpha\left(\nu_{+}z_{+}u_{+}+\nu_{-}\left\vert z_{-}\right\vert u_{-}\right)EF \\ =Sc\alpha\nu_{+}z_{+}\left(u_{+}+u_{-}\right)EF \end{matrix}$ | (S3) |
| --- | --- |

To measure the contribution of diverse ions to conductivity, the ratio of the current formed by ions to the total current is defined as the ion transference number *t_B_*

| $t_{B}=\frac{I_{B}}{I}$ | (S4) |
| --- | --- |

From Eq. S3 is easy to derive

| $t_{\text{+}}=\frac{u_{\text{+}}}{u_{\text{+}}+u_{-}}\begin{matrix} & \text{，} & \end{matrix}t_{-}=\frac{u_{-}}{u_{\text{+}}+u_{-}}$ | (S5) |
| --- | --- |

From Eqs. (6) and (7), the relationship between conductivity and current in the conductivity cell is

| $\kappa=\frac{I}{ES}$ | (S6) |
| --- | --- |

Substituting Eq. S3 into S6, we get

| $\kappa=c\alpha\nu_{+}z_{+}\left(u_{+}+u_{-}\right)F$ | (S7) |
| --- | --- |

Substituting Eq. S7 into S5, the relationship between ion mobility and conductivity can be expressed as

| $u_{\text{+}}=\frac{t_{\text{+}}\kappa}{c\alpha v_{+}z_{+}F}\begin{matrix} & , & \end{matrix}u_{-}=\frac{t_{-}\kappa}{c\alpha v_{-}\left\vert z_{-}\right\vert F}$ | (S8) |
| --- | --- |

Therefore, we know that conductivity is directly proportional to ion mobility. Different ions contribute to ion mobility, collectively affecting the solution's conductivity. When selecting solutes for conductive liquids, the primary consideration is to avoid extreme pH values, which indicate high concentrations of H^+^ (H_3_O^+^) or OH^-^. H^+^ (H_3_O^+^) and OH^-^ have unique rapid chain proton transfer mechanisms in water or polyhydric polar solutions, leading to high ion mobility and intensified electrolytic reactions. This explains why water has high chemical activity. Although pure water has a pH of 7 with limited hydrolysis, the free H^+^ (H_3_O^+^) and OH^-^ generated during dielectric failure exacerbate hydrolysis. Li^+^ has a strong electric field effect on water molecules, forming a tight hydration layer and increasing its migration resistance in the solvent. Aside from Li^+^, common metal cations, halide anions, and non-metal oxide anions have small ionic sizes, resulting in high ion mobility in water. In contrast, organic ions have larger sizes and lower ion mobility, making organic salts suitable additives in polar solvents.

Additionally, for electrowetting devices driven by AC voltage, the total current response to a time-varying electric field is given by equation S9:

| $\overrightarrow{j_{\text{tot}}}=\kappa\overrightarrow{E}+\varepsilon_{0}\varepsilon_{\text{r}}\frac{\partial\overrightarrow{E}}{\partial t}$ | (S9) |
| --- | --- |

For a periodic electric field *E*(*t*)=*E*_0_ exp(*iωt*), the dielectric response combines the system's dissipative in-phase and capacitive out-of-phase responses, resulting in a complex current and complex conductivity

| $\overrightarrow{j_{\text{tot}}}=\kappa^{*}\overrightarrow{E}$ | (S10) |
| --- | --- |
| $\kappa^{*}=\kappa+i\omega\varepsilon_{0}\varepsilon_{\text{r}}$ | (S11) |

where *ω* is the frequency of the periodic electric field.

Therefore, a characteristic relaxation time can be determined

| $\tau_{\text{el}}=\frac{\varepsilon_{0}\varepsilon_{\text{r}}}{\kappa}$ | (S12) |
| --- | --- |

For *ω*≪*τ*_el_^-1^, the response is controlled by an Ohmic response, and the electrolyte behaves as a perfect conductor. Conversely, for *ω*≫*τ*_el_^-1^, the electrolyte behaves as a dielectric. Thus, we can calculate the required solution conductivity based on the frequency of the AC voltage applied to the electrowetting device and subsequently determine the concentration of the electrolyte solute needed.

In the Section Theory and Principle and Supplementary Material S1.1, during the derivation of the mechanism to suppress dielectric failure by rational selection of conductive liquid materials, we note that when driving the electrowetting liquid devices with AC voltages, we can infer the required conductivity of the conductive liquid based on the frequency of the AC voltage used to drive the device. Several studies suggest that higher-frequency AC voltages can offer various benefits for electrowetting actuation, such as reducing liquid surface oscillations and jitter^1–3^. In our experiments, we employ a 1k Hz AC voltage. According to Eq. S12 in Supplementary Material S1.1, which provides the characteristic relaxation time *τ*_el_ for electrowetting devices driven by AC voltages at *ω*=1k Hz frequency, in order to ensure that the reciprocal of the characteristic relaxation time *τ*_el_^-1^ ≫ 1k Hz, we can calculate that the conductivity of the conductive liquid needs to be greater than ~29 μS. Furthermore, based on the analysis of the electrolytic reactions in Section 3.1.3, replacing the electrolyte with substances like tetrabutylammonium acetate, which are less prone to participating in reactions, may yield better results.

**S2 Details of liquid parameters and electrowetting characteristics**

**S2.1 Details of liquid parameters**

Since we use non-aqueous polar organic solvents to develop the conductive liquid, we must select a reliable, strongly non-polar liquid to develop the insulating liquid to avoid mixing the two electrowetting phases. ISOPAR™ fluid, a hydrocarbon compound derived from petroleum-based raw materials through hydrogenation in the presence of a catalyst, is primarily composed of isoparaffins and cycloparaffins. It is an excellent solvent due to its strong non-polarity. However, the density of ISOPAR™ fluid is relatively low, below 1 g∙cm^-3^, and the refractive index is also relatively low. Therefore, to match the density with the conductive liquid and increase the refractive index difference between the biphasic liquids, we add halogenated aromatic compounds with higher density to ISOPAR™ V fluid. In this study, we use PB to enhance the performance of the insulating liquid.


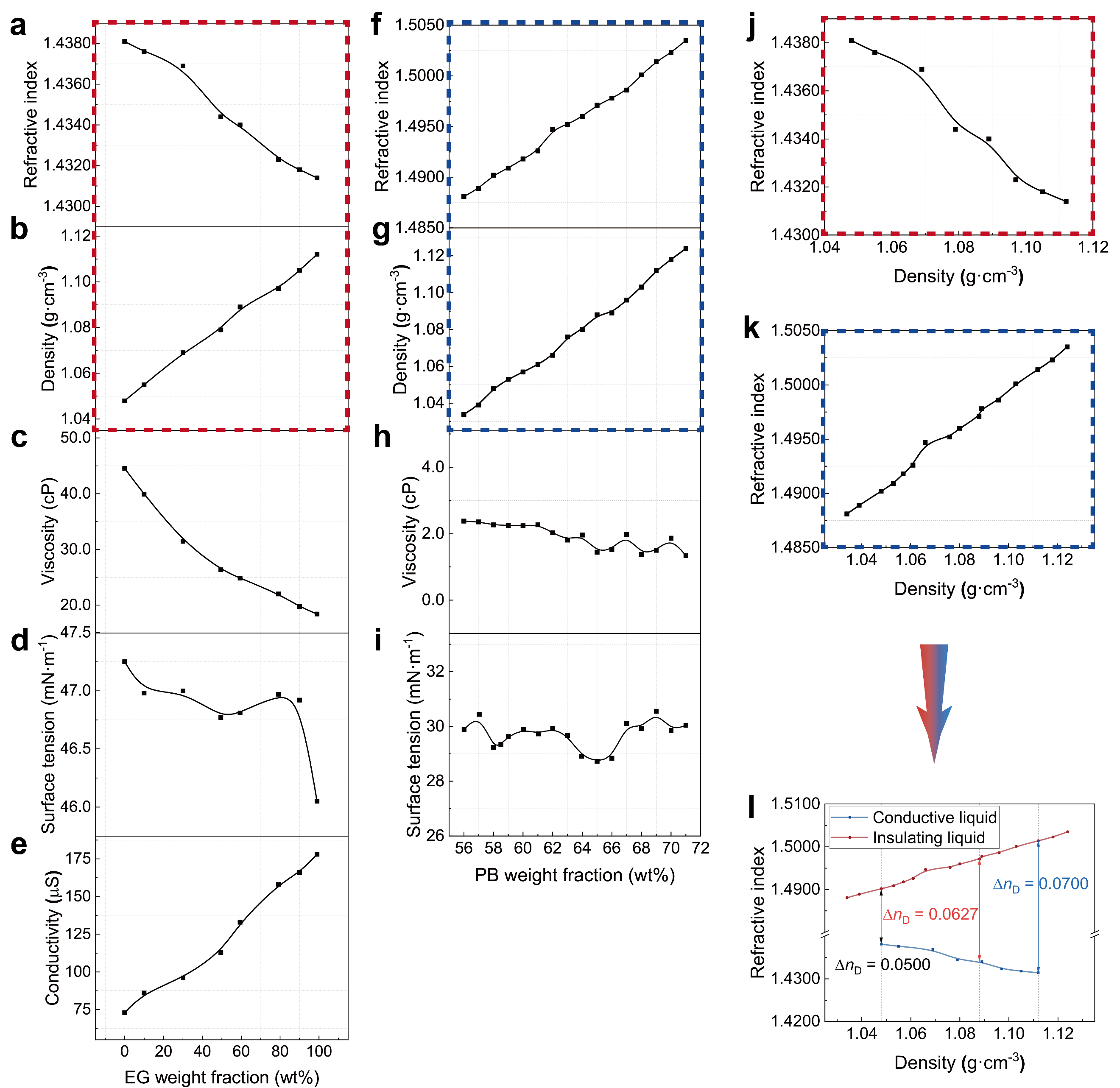


**Fig. S1 Physical properties of the biphasic liquids. a-e** Relationship between EG weight fraction and refractive index, density, viscosity, surface tension, and conductivity of the conductive liquid, respectively. **f-i** Relationship between PB weight fraction and refractive index, density, viscosity, surface tension, and conductivity of the insulating liquid, respectively. **j** Relationship between density and refractive index of the conductive liquid. **k** Relationship between density and refractive index of the insulating liquid. **l** Relationship between density and refractive index of the biphasic liquids.

We prepare a series of mixtures with different PB and ISOPAR™ V fluid concentrations, namely PBI series insulating liquids, and measure their refractive index, density, viscosity, and surface tension. The test results of PBI and EGG series liquids are shown in Fig. S1 collectively. In the preparation of the EGG and PBI series, we use an analytical balance (Type of JA203P, Changzhou XINGYUN Electronic Equipment Co., Ltd, China) to weigh the mass of each liquid raw material, a float-type electronic densimeter (Type of DK-300S, Xiamen Qunlong Instrument Co., Ltd, China) to measure the liquid density, an Abbe refractometer (Type of WYA-2S, Shanghai INESA Physico-Optical Instrument Co., Ltd., China) to measure the refractive index under 589.3nm, a digital viscometer (Type of NDJ-9S, Shanghai Fangrui Instrument Co., Ltd, China) to measure liquid viscosity, and a platinum plate method surface tensiometer (Type of BZY-201, Shanghai Fangrui Instrument Co., Ltd, China) to test the surface and interfacial tension. Additionally, we use a conductivity pen (Type of SX-650, Shanghai Sanxin Peirui Instrument Co., Ltd, China) to measure the electrical conductivity of the liquids. Figs. S1a-e show the refractive index, density, viscosity, surface tension, and conductivity of the EGG series liquids. Figs. S1f-i show the refractive index, density, viscosity, and surface tension of the PBI series liquids. Since the result of measuring the conductivity of the PBI series insulating liquids is zero, no corresponding graph is plotted. Fig. S1j shows the relationship between refractive index and density for the EGG series liquids, Fig. S1k shows the same relationship for the PBI series liquids, and Fig. S1l displays the refractive index difference when the densities of the biphasic liquids match.


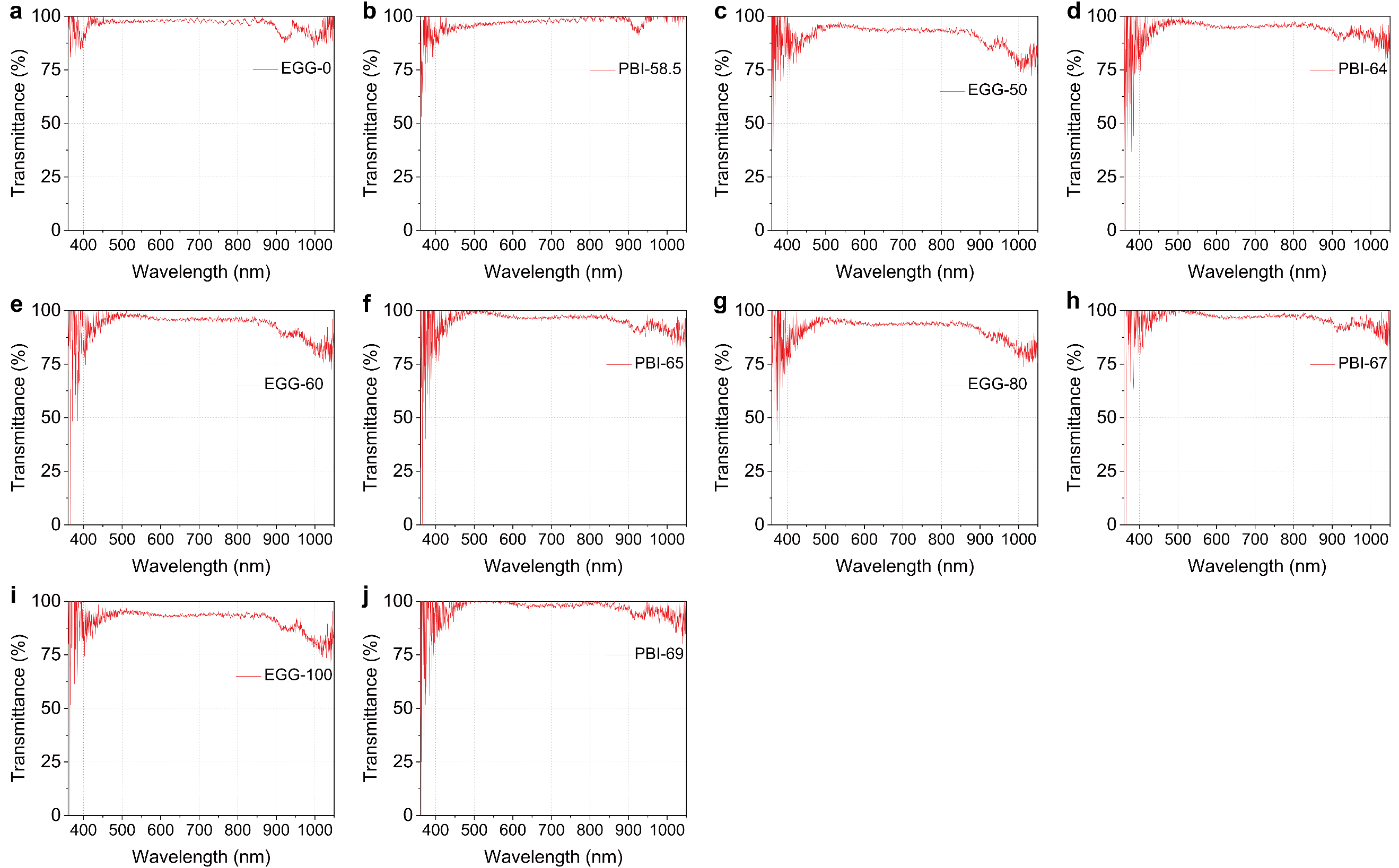


**Fig. S2 Transmittance of the biphasic liquids in the EGG series lenses. a, c, e, g, i** Transmittance of the EGG series liquids. **b, d, f, h, j** Transmittance of the PBI series liquids.

Additionally, we use a spectrometer (Type of Aurera 4000, CNI Co., Ltd., China) to test the transmittance of all the liquids above. The spectrometer has an effective measurement wavelength range of 400 nm to 1000 nm. The results are shown in Fig. S2. In the entire EGG series liquids, the transmittance in the visible light band is greater than 90%, and in the near-infrared band, it is greater than 70%. In the entire PBI series liquids, the transmittance in the visible light band is greater than 93%, and in the near-infrared band, it is greater than 80%.

**S2.2 Details of electrowetting characteristics of EGG series liquids**


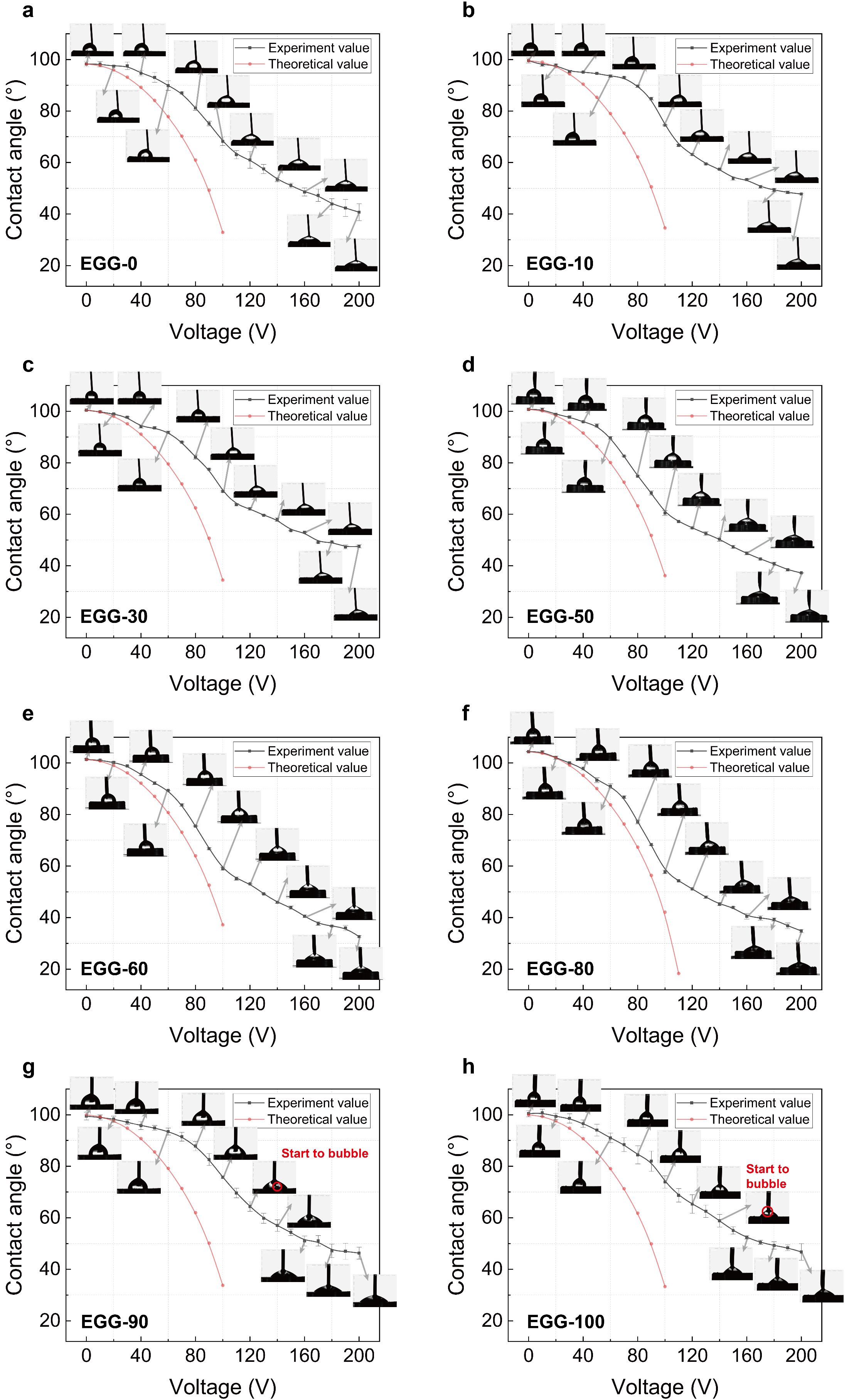


**Fig. S3 Variation of the contact angle with voltage for the EGG series conductive liquids: experimental values vs. theoretical values.**

We measure the electrowetting characteristics of the EGG series liquids in air using the setup shown in Figs. 4a-b on a contact angle measuring instrument (Type of JCY-2, Shanghai Fangrui Instrument Co., Ltd, China). Add ~10 μL of liquid onto the flat plate. Specifically, we arrange the setup on a three-dimensional precision displacement platform, using a long-focus camera and CCD to capture the changes in the contact angle of the droplet under different applied voltages. The contact angles of the droplets are measured using the three-point method in the measurement software MindVision Technology. The experimental results are compared with theoretical values, as shown in Fig. S3. Fig. S3 shows the relationship between contact angle and voltage for single-phase droplets of the EGG series liquids, and the theoretical values is calculated directly from Eq. (1). The accuracy of the theoretical values is influenced by the method and precision of surface tension measurements of the EGG series liquids. In the experiment, a platinum plate surface tensiometer with a precision of 0.01 mN∙m^-1^ was used. Compared with the more accurate droplet shape method, the platinum plate method may introduce a slight underestimation error due to the finite thickness of the platinum plate and the non-zero contact angle formed by liquid adsorption during the test. Thus, the theoretical contact angles calculated at the same voltage may appear slightly lower. For the measured values, the roughness of the processed metal plate also contributes to slightly higher contact angles at the same voltage. Furthermore, the actual electrowetting effect exhibits contact angle saturation. Our tests show that the EGG series liquids reach a saturation contact angle at 110 V to 120 V, aligning closely with our calculated theoretical values. Overall, the contact angle-voltage relationship of the EGG series liquids shows a consistent trend with the theoretical values and has the smallest deviation before saturation voltage.

**S2.3 Details of dielectric failure experiment**

We test the pH values of the conductive liquids after dielectric failure and compare them with the pH values of pure water, the conductive liquid, and the insulating liquid itself, as shown in Fig. S4. We fill a liquid lens with EGG-100 and a density-matched PBI-100 (insulating liquid), applying a 1k Hz, 200 V AC voltage for 10 minutes. After observing no new bubbles, we extract the conductive liquid from the lens, separate it from the insulating liquid, and use Merck® pH-indicator strips to detect the pH of the electrolyzed conductive liquid. Before the dielectric failure, the pH values of the conductive and insulating liquids are similar to pure water. After dielectric failure, the pH of the conductive liquid ranges between 7.7 and 7.9, indicating weak alkalinity.


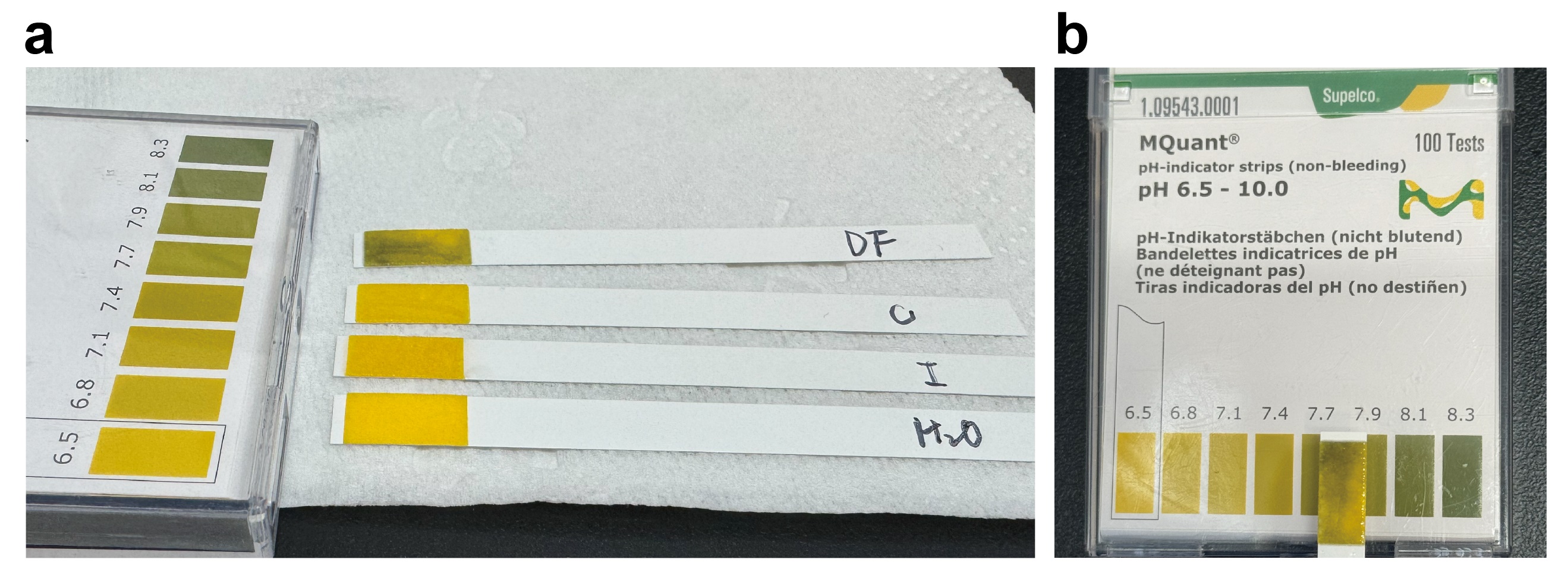


**Fig. S4 PH values of the liquids.** **a** Comparison of pH values: from top to bottom are the EGG-100 after dielectric failure, the original EGG-100, the PBI-100, and deionized water, respectively. **b** PH value of the EGG-100 after dielectric failure compared with the pH indicator strips.

Here, we provide an explanation for why the electrodes do not participate in the electrolysis reaction. In electrowetting experiments, one electrode is specially treated with a coating, while the other is in direct contact with the conductive liquid. In our experiments, the electrodes used are as follows: (1) for droplet experiments on the planar electrode, copper (Cu) is coated, while the platinum electrode directly contacts the conductive droplet; (2) for the lenses we made, aluminum (Al) is coated, while indium tin oxide (ITO) directly contacts the conductive liquid. However, in previous studies, few researchers doubted the involvement of electrodes in the reaction, primarily because no corresponding phenomena were observed. In some specific solution environments, such as in direct current electrolysis cells with high Cl^-^ content, ITO has been observed to be reduced to In and Sn metals^4,5^. However, we consider the electrodes to be inert in electrowetting devices driven by AC, especially at high frequencies. When metals participate in electrolytic cell reactions, the products must be metal ions. Copper metal loses electrons at the anode to form Cu^2+^; aluminum metal forms Al^3+^. However, in the solution environment of our experiments, we have never observed the liquid turning blue, green, or yellow, nor have we observed the formation of the white or blue precipitate. Therefore, we believe that the electrolytic reactions of Cu and Al electrode do not occur. On the other hand, the Al electrode is reported to have a self-healing phenomenon, where after the formation of Al^3+^ through electrolysis, it combines with water molecules to form a dense Al_2_O_3_ layer, thereby isolating a portion of the electrode^6^. In summary, the selection of electrodes described in this study does not affect the analysis of electrolytic reactions of the solution during dielectric failure.

To further validate the suppressed effect of the EGG series on dielectric failure, we conduct multiple cycle tests after measuring the relationship between contact angle and applied voltage for the EGG series conductive liquids. We measure the recovery of the initial contact angle of the droplets and the contact angles at 80 V and 100 V. The experimental results are shown in Figs. S5 and S6. When measuring the initial contact angle, we observe both the left and right contact angles of the droplets. The maximum deviation between these angles reflects whether the recovery deviation is due to changes in the liquid's inherent properties or to interfacial inhomogeneities. During the droplet recovery process after voltage removal, the absence of electric field force allows the droplet to restore to the initial contact angle relying on its surface tension. However, the roughness of the plate surface may affect the recovery of the droplet contact angle to some extent. As shown in Fig. S5, in the five-cycle measurements of the initial contact angle of EGG series liquids, both the left and right contact angles deviated to varying degrees from the first measurement when the droplet is dropped on the plate, and most of these deviations are decreases. This is because during the process of droplet recovery to the initial contact angle, the direction of droplet triple line movement is opposite to the direction of the frictional force on the plate surface. Some larger defects on the plate surface may also act as rivets on the triple line, causing a significant decrease in the initial contact angle during measurement.

For EGG-0 to EGG-80, which do not experience dielectric failure, the contact angle recovery deviation is less than 11.2 °, with a maximum left-right deviation of less than 9.8 °. The distribution of these deviations is random and does not increase with repeated measurements. EGG-60 shows the best performance, with a maximum recovery deviation of 4 ° and a maximum left-right deviation of 1.3 °. For EGG-90 and EGG-100, which experience dielectric failure, the maximum recovery deviation of the initial contact angle is decreased by 9.5 ° and 19.9 °, respectively, with maximum left-right deviations of 6 ° for EGG-90 and 4.8 ° for EGG-100. The initial contact angle recovery deviation of EGG-90 is not much different from that of EGG-0 to EGG-80 in numerical terms, while EGG-100 shows a large deviation, confirming our previous conclusion that liquids with higher conductivity exhibit faster electrochemical reaction rates and more pronounced phenomena after dielectric failure. Furthermore, as analyzed earlier, the electrochemical reactions that occur during dielectric failure lead to changes in ion concentration in the liquid, an increase in pH, a decrease in surface tension, and the inability of the droplet to return to the initial contact angle. Additionally, as the number of cycles increases, the magnitude of the deviation increases, reflecting the time-dependent nature of dielectric failure as a phenomenon in electrowetting. The effects of dielectric failure accumulate over time. However, even for EGG-100, which experiences the most severe dielectric failure, the values of the left and right contact angles stabilize after the third cycle, and the deviation almost ceases to increase.


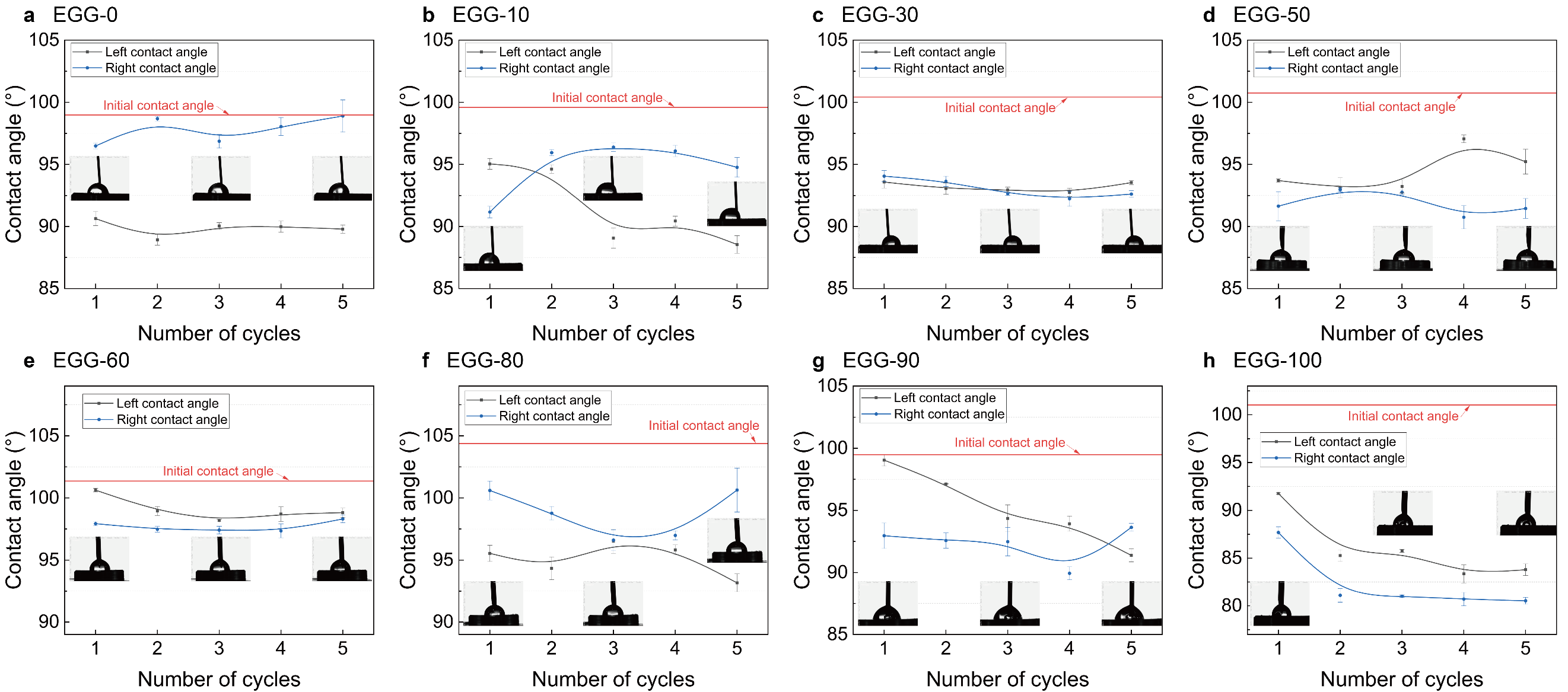


**Fig. S5** **Initial contact angle after each power-off in the cycles of EGG series liquids.**

We also conduct multiple measurements of the contact angle of the EGG series liquids under applied voltages of 80V and 100V, as shown in Fig. S6. All EGG series liquids are consistent, with deviations ranging from -5.6 ° to +3.5 °. For EGG-90 and EGG-100, where dielectric failure occurs, the droplet's response to voltage remains the same as when dielectric failure does not occur. As analyzed earlier, dielectric failure does not occur continuously in the EGG series liquids, and the materials involved in electrochemical reactions during dielectric failure are not the main components of the conductive liquid. The main components of the conductive liquid are numerous non-aqueous, strongly polar molecules that do not participate in electrochemical reactions. They can continuously rearrange under external electric fields through polarization effects, allowing the liquid to be driven. Moreover, based on our experimental results, the electrowetting response of the droplet is almost identical to that before dielectric failure. According to the cyclic test results of the initial contact angle, we have verified the inhibitory effect of the EGG series liquids on dielectric failure.


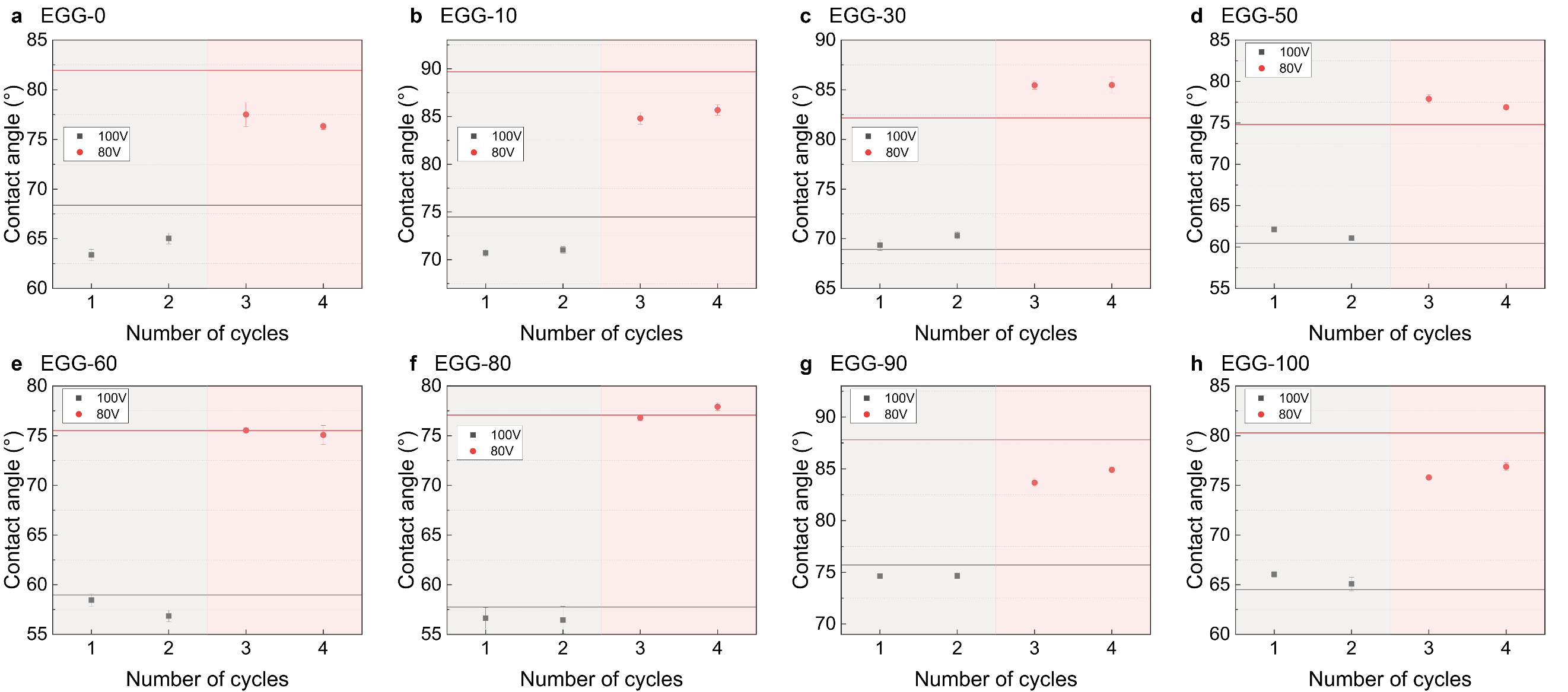


**Fig. S6 Contact angle at 80V and 100V after each power-on in the cycles of EGG series liquids.**

**S3 Details of EGG series electrowetting liquid lenses**

**S3.1 Design and fabrication**

To achieve both a wide field of view and a large optical focal variation range, we need to compress the lens thickness as much as possible while avoiding the dynamic range limitation caused by an excessively thin thickness at the three-phase line. To ensure the three-phase line moves on the dielectric layer within the applied voltage range and that the liquid-liquid interface center does not touch the upper or lower glass during voltage application and removal, we simulate the height of the meniscus center from the lower glass and the height of the three-phase line from the lower glass over time using COMSOL Multiphysics®. The simulation results are shown in Fig. S7. The simulated lens has a diameter of 10 mm, filled with EGG series liquids and a density-matched PBI series insulating liquid, with an applied voltage of 60 V. When the voltage is removed, the liquid-liquid interface drops from a contact angle of 90 °. According to the simulation results in Fig. S7, the main limitation of lens thickness compression is the meniscus oscillation. To ensure stable operation of the liquid lens, we reserve some height margin and design the liquid lens thickness to be 6 mm, with a 1:1 ratio of conductive liquid to insulating liquid.


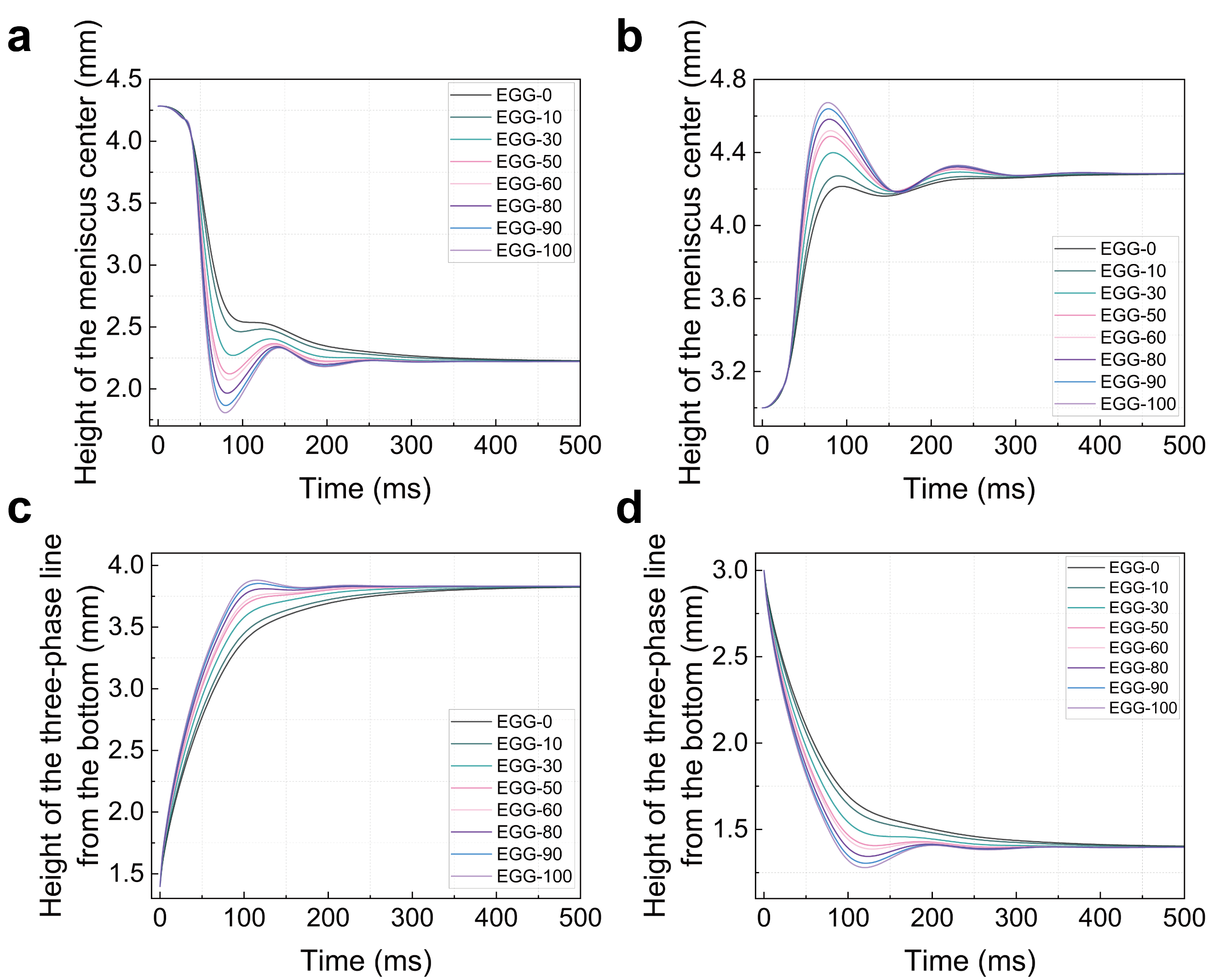


**Fig. S7 Variation of the height of the meniscus center and the three-phase line from the lower electrode with time for the 10mm lens.** **a** Variation of the height of the meniscus center with time when a 60 V voltage is applied. **b** Variation of the height of the meniscus center with time when the voltage is removed. **c** Variation of the height of the three-phase line from the lower electrode with time when a 60 V voltage is applied. **d** Variation of the height of the three-phase line from the lower electrode with time when the voltage is removed.

After determining the mechanical dimensions of the lens, we use advanced computer numerical control precision machining technology to manufacture the lens components. Specifically, we finely polish the metal electrode to achieve an inner wall surface roughness of Ra0.15. The upper electrode is made of aluminum, with a dielectric layer (ParyleneC, 3 μm) and a hydrophobic layer (Teflon AF2400, 100 nm) sequentially coated on top. The lower electrode is ITO glass, which also serves as the lower window glass. After bonding and curing the upper electrode, shim, and lower electrode with high-strength two-component epoxy resin, we use a pipette to precisely inject the biphasic liquids based on the calculated cavity volume. We cover the insulating liquid with the upper glass using the oil immersion method to avoid bubble formation. Finally, we seal the liquid lens by filling the reserved groove between the upper glass and the upper electrode with high-strength two-component epoxy resin. The flexible electrode is designed to fit directly over the outer diameter of the upper electrode and gasket, and it is glued to the upper and lower electrodes with high-strength conductive silver adhesive. The liquid lens driver is self-developed based on STM32G030F6P6 and provides sufficient driving voltage for the liquid lens with a voltage adjustment step of ~0.2 V. The fabricated lens samples and component details are shown in Fig. S8.


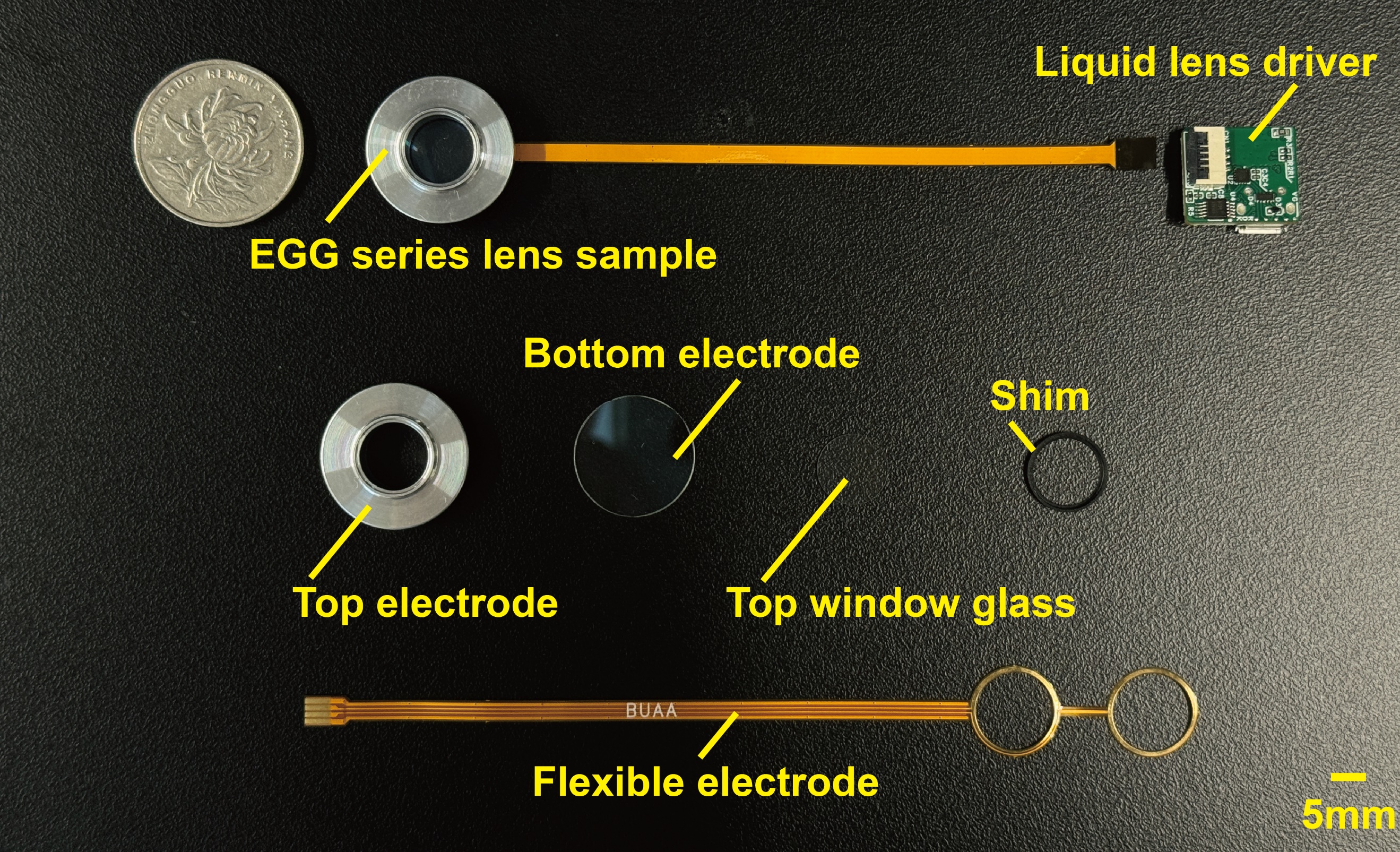


**Fig. S8 Samples and components of the EGG series lens.**

**S3.2 Details of liquid lens parameters and performance experiment**

We combine EGG-0, EGG-50, EGG-60, EGG-80, and EGG-100 with their density-matched PBI insulating liquids to form five groups of biphasic liquids. They are filled into cylindrical liquid lens cavities with a diameter of 10 mm and a height of 6 mm. The parameters of the liquids used in the five liquid lenses are listed in Table S1, where "C" denotes the conductive liquid and "I" denotes the insulating liquid.

**Table S1 Liquid lens filling liquid parameters**

| Parameter  Description | | Refractive index  (D light) | Density  (g∙cm^-3^) | Viscosity  (cP) | Interfacial tension  (mN∙m^-1^) |
| --- | --- | --- | --- | --- | --- |
| EGG-0 | C | 1.4381 | 1.048 | 44.56 | 11.54 |
|  | I | 1.4902 | 1.048 | 2.27 |  |
| EGG-50 | C | 1.4355 | 1.079 | 26.38 | 11.68 |
|  | I | 1.4960 | 1.080 | 1.96 |  |
| EGG-60 | C | 1.4344 | 1.087 | 24.87 | 11.42 |
|  | I | 1.4971 | 1.087 | 1.45 |  |
| EGG-80 | C | 1.4323 | 1.097 | 22.02 | 11.70 |
|  | I | 1.4986 | 1.096 | 1.98 |  |
| EGG-100 | C | 1.4314 | 1.112 | 18.43 | 11.36 |
|  | I | 1.5014 | 1.112 | 1.50 |  |


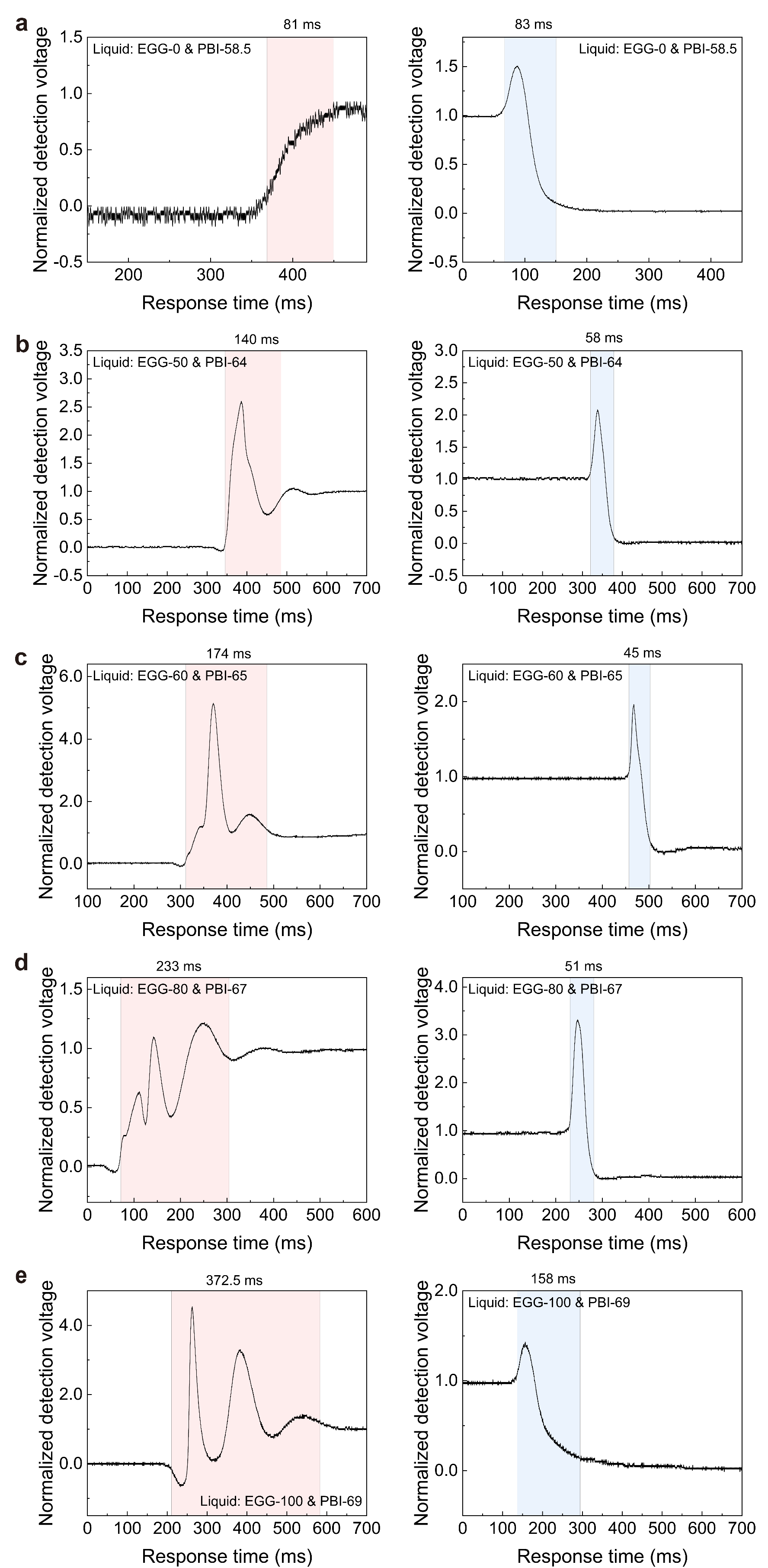


**Fig.S9 Response time of EGG series liquid lenses. a** Rise and fall response time of EGG-0 liquid lenses. **b** Rise and fall response time of EGG-50 liquid lenses. **c** Rise and fall response time of EGG-60 liquid lenses. **d** Rise and fall response time of EGG-80 liquid lenses. **e** Rise and fall response time of EGG-100 liquid lenses.

We also test the response time of the five liquid lenses produced. A 632.8 nm helium-neon laser (Type of DH-HN250, Daheng Co., Ltd, China), polarizers, attenuators, a Si amplified detector (Type of PDF 10 A/M, Thorlabs, Inc., America), an aperture, and an oscilloscope are used to form the test system. The Si-amplified detector converted the light signal into a voltage signal displayed on the oscilloscope. We test the response of the lenses from no voltage applied to 60 V applied with a 1kHz sinusoidal voltage signal, normalized the voltage signal display on the oscilloscope, and take the time between 10% and 90% of the stable state on the oscilloscope as the response time of the lenses produced. The results are shown in Fig. S9. Based on the calculations, the rise response time of the liquid lenses filled with EGG-0, EGG-50, EGG-60, EGG-80, and EGG-100 are 81 ms, 140 ms, 174 ms, 233 ms, and 372.5 ms, respectively, while the fall response times are 83 ms, 58 ms, 45 ms, 51 ms, and 158 ms, respectively. In this experiment, a low-viscosity insulating liquid is used, indicating that all lenses are in a state of under-damped oscillation. The rise time of the lenses is related to the viscosity of the biphasic liquids; the lower the viscosity tested, the more pronounced the oscillation and the longer the response time. The fall time of the lenses is influenced more by the properties of the liquids and the lens itself, such as the viscosity of the liquid and the interfacial tension, as well as the roughness of the electrodes, dielectric, and hydrophobic layers. It can be observed that the lenses filled with EGG-50 to EGG-80 still have good fall times under a 10mm aperture.

For large-aperture electrowetting liquid lenses, improving response time is a challenge. Our previous work shows promising results with EGG-0 combined with a low-viscosity insulating phase. Still, in this study, both the EGG series and the low-viscosity insulating phase exhibit longer response time due to under-damped oscillation under a 10 mm aperture. Our future work will focus on optimizing the insulating phase. Additionally, we observe the influence of the roughness of the inner walls of the electrowetting liquid lens on its performance, including response time, optical power, and dielectric failure performance. We will delve deeper into this aspect in our future work to enhance the overall performance of our lenses.

The imaging experiments are presented in Section 3.2. The focal ratio of the solid lens group (M12HF12, Shenzhen Jinghang Technology Co., Ltd) is 2.8, with a focal length of 12 mm and a mechanism length of approximately 19 mm. The image sensor uses a photosensitive chip of type Sony IMX178, with a sensor area size of 1/1.8” and a pixel size of 2.4 μm. For each liquid lens-based zoom imaging system, in addition to the experimental results of the zoom imaging system focusing at 0 V, 60 V, and 70 V, we also capture the continuous zoom process of the system as the applied voltage varies from 0 V to 70 V, with the camera gradually shifting focus from a distant object to a near object, as shown in Figs. S10 and S11.


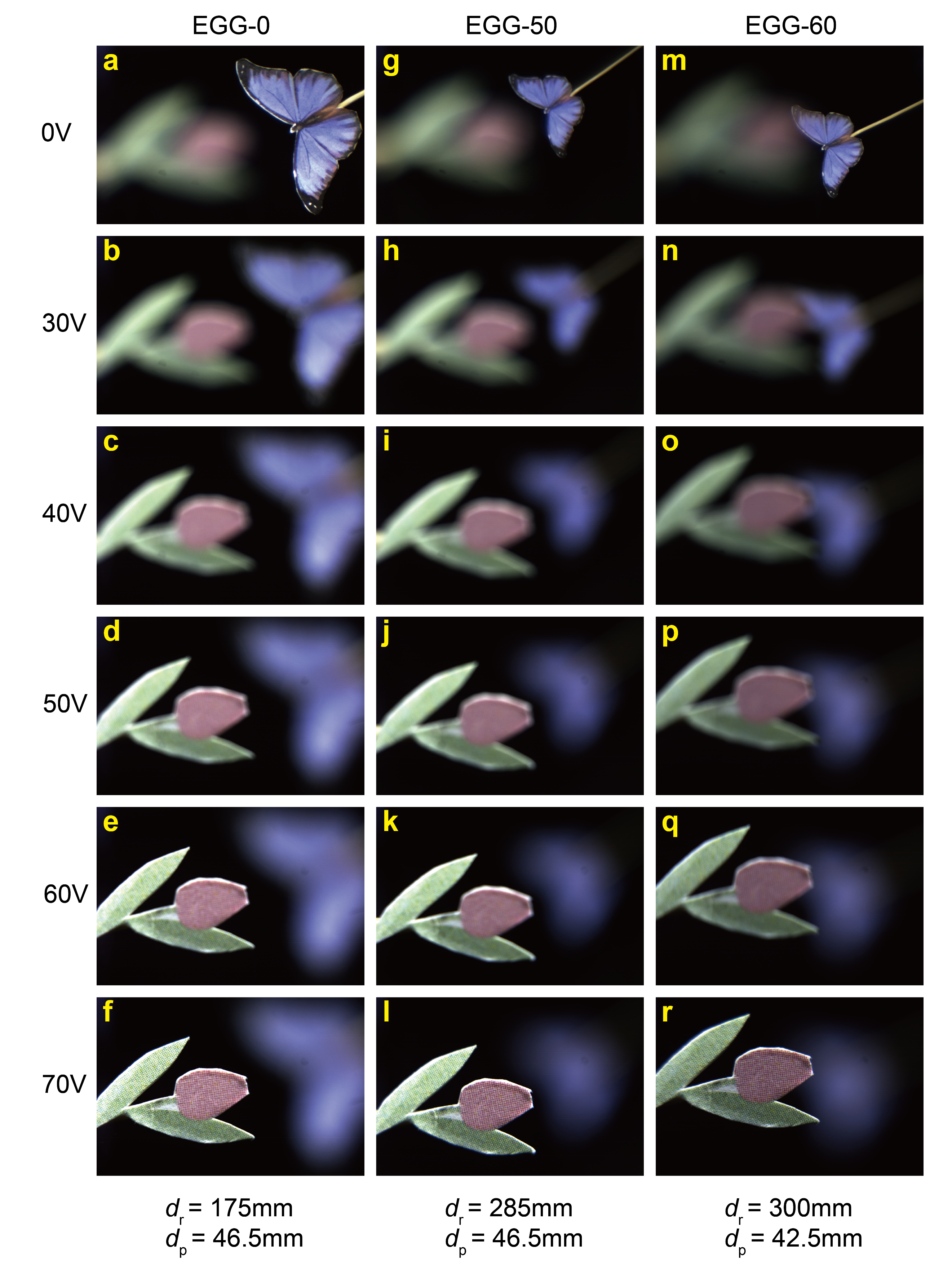


**Fig. S10 Continuous zooming experimental results of the zoom imaging system with EGG-0, EGG-50, and EGG-60 liquid lenses. a-f** The continuous zoom process of the EGG-0 liquid lens-based zoom imaging as the applied voltage varies from 0 V to 70 V. **g-l** The continuous zoom process of the EGG-50 liquid lens-based zoom imaging as the applied voltage varies from 0 V to 70 V. **m-n** The continuous zoom process of the EGG-60 liquid lens-based zoom imaging as the applied voltage varies from 0 V to 70 V.


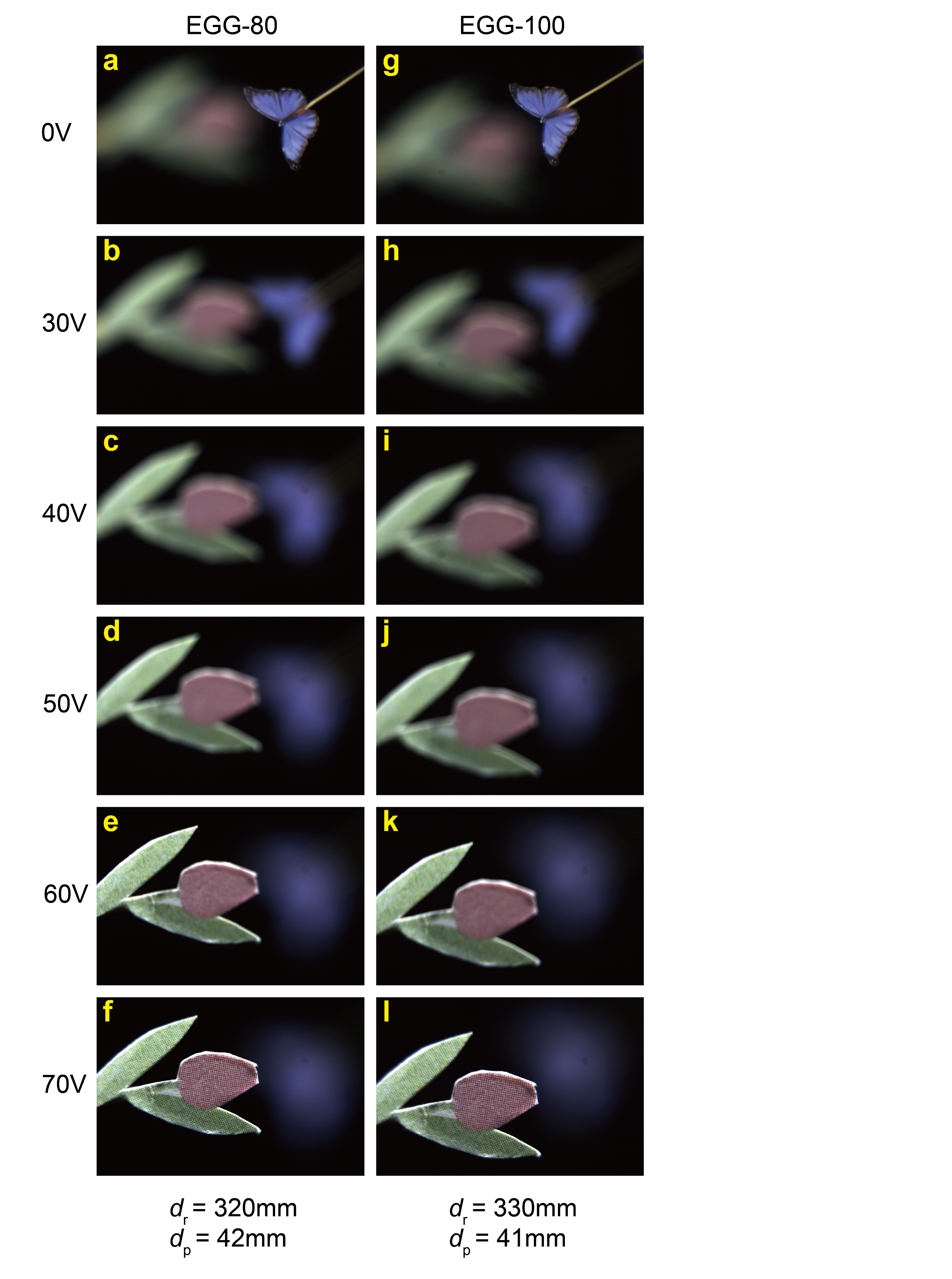


**Fig. S11 Continuous zooming experimental results of the zoom imaging system with EGG-80 and EGG-100 liquid lenses. a-f** The continuous zoom process of the EGG-80 liquid lens-based zoom imaging as the applied voltage varies from 0 V to 70 V. **g-l** The continuous zoom process of the EGG-100 liquid lens-based zoom imaging as the applied voltage varies from 0 V to 70 V.

**S3.3 Details of application experiment** **of the EGG-60 electrowetting liquid lens**

Figs. S12a, b, i, j, Figs. S12c, d, k, l, Figs. S12e, f, m, n, and Figs. S12g, h, o, p show experiment results in which the distances between the camera and Lens II are 540 mm, 440 mm, 340 mm, and 240 mm, respectively. This can be seen from the holographic reconstructed images in the blue boxes in Figs. S12a-b that when the “flower” and “deer” are focused, respectively, the edges of the “flower” and the horns of the “deer” can not be completely reconstructed. This is caused by the small aperture of the commercial A-39N0 liquid lens. When the distance between the camera and lens II changes, it can be seen from Figs. S12c-d, Figs. S12e-f, and Figs. S12g-h that the edges of the “flower” and the horns of the “deer” can not be completely reconstructed, which indicates that the commercial A-39N0 liquid lens can not efficiently achieve holographic 3D reconstruction. From the holographic reconstructed images in the red boxes in Figs. S12i-j, it can be found that the edge of the “flower” and the horn of “deer” can be reconstructed completely when the “flower” and “deer” are focused, respectively. This proves the advantage of the proposed EGG-60 liquid lens in terms of aperture. When the distance between the camera and lens II changes, it can be seen from Figs. S12k-l, Figs. S12m-n, and Figs. S12o-p that the edges of the “flower” and the horns of the “deer” can be reconstructed completely. Experiments prove that the proposed EGG-60 liquid lens can guarantee the quality of the holographic reconstructed image as well as realize the position change of the holographic reconstructed image.


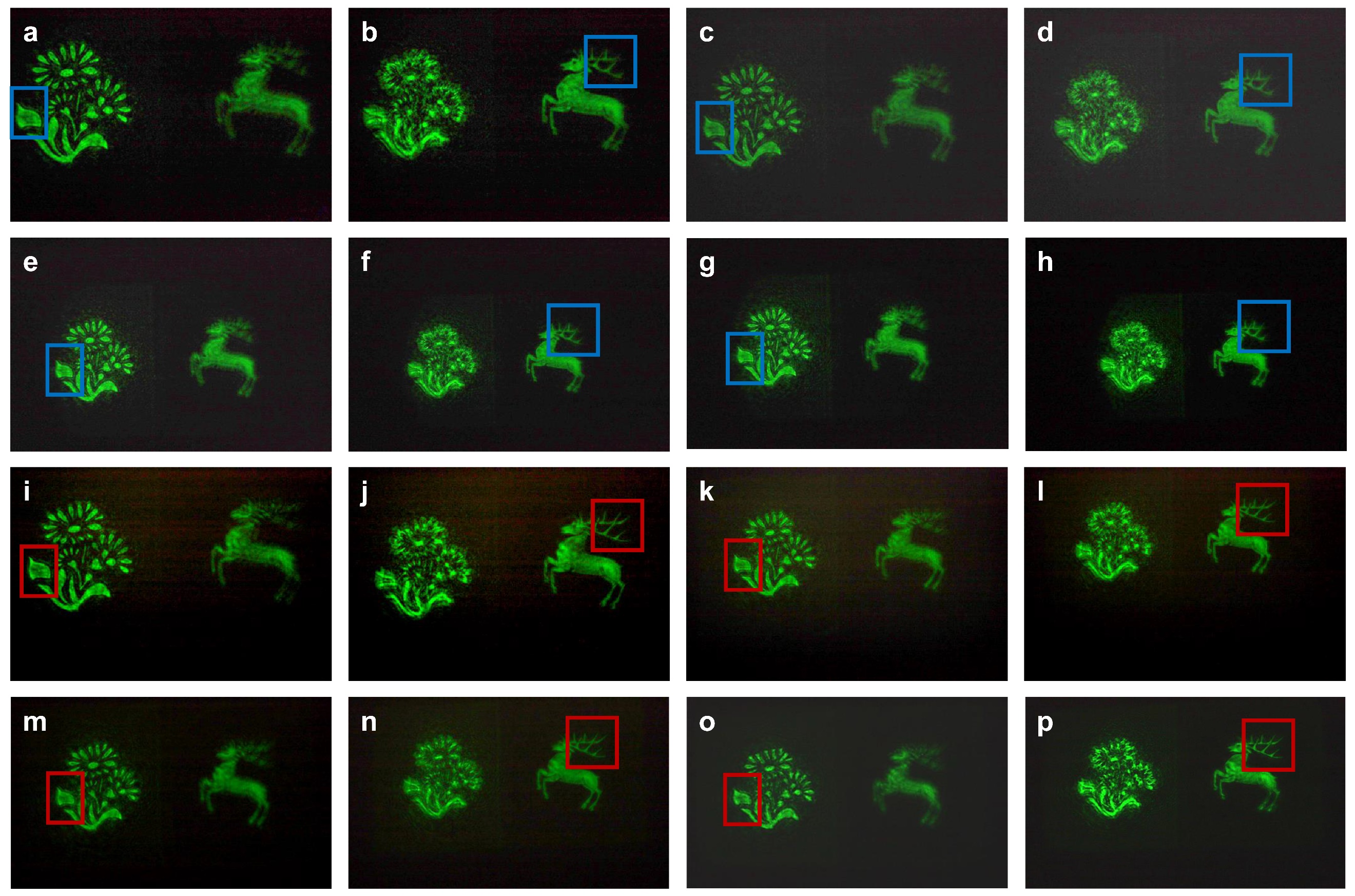


**Fig. S12 Comparison of the holographic reconstructed images at different reconstruction positions. a-h** Experimental effects using the commercial A-39N0 liquid lens. **i-p** Experimental effects using the proposed EGG-60 liquid lens.

**References**

1. Oh, J. M., Ko, S. H. & Kang, K. H. Shape oscillation of a drop in ac electrowetting. *Langmuir* **24**, 8379–8386 (2008).

2. Murade, C. U., Van Der Ende, D. & Mugele, F. High speed adaptive liquid microlens array. *Opt. Express* **20**, 18180–18187 (2012).

3. Wang, L.-M., Zhao, R., Liang, Z.-C., Zhang, J. & Kou, S.-F. Effect of frequency on droplet actuation in reverse electrowetting. *J. Micromech. Microeng.* **33**, 035003 (2023).

4. Zhou, R. *et al.* Experimental study on the reliability of water/fluoropolymer/ITO contact in electrowetting displays. *Results Phys* **12**, 1991–1998 (2019).

5. Liu, L., Yellinek, S., Valdinger, I., Donval, A. & Mandler, D. Important implications of the electrochemical reduction of ITO. *Electrochimica Acta* **176**, 1374–1381 (2015).

6. Dhindsa, M., Heikenfeld, J., Weekamp, W. & Kuiper, S. Electrowetting without electrolysis on self-healing dielectrics. *Langmuir* **27**, 5665–5670 (2011).
